# MicroRNA-7641 is a regulator of ribosomal proteins and a promising targeting factor to improve the efficacy of cancer therapy

**DOI:** 10.1038/s41598-017-08737-w

**Published:** 2017-08-21

**Authors:** Abu Musa Md. Talimur Reza, Yun-Jung Choi, Yu-Guo Yuan, Joydeep Das, Hideyo Yasuda, Jin-Hoi Kim

**Affiliations:** Department of Stem Cell and Regenerative Biotechnology, Humanized Pig Research Centre (SRC), Konkuk University, Seoul, 143-701 Republic of Korea

## Abstract

Many diseases, including myocardial infarction, autoimmune disease, viral diseases, neurodegenerative diseases, and cancers, are frequently diagnosed with aberrant expression of microRNAs (miRNAs) and their allied pathways. This indicates the crucial role of miRNAs in maintaining biological and physiological processes. miR-7641 is a miRNA whose role in disease has not been fully investigated. In the present study, we investigated the expression pattern of miR-7641 and its target genes in different cancer cells, as well as in clinical cancer patients. Our data confirmed *RPS16* and *TNFSF10* as two direct targets of miR-7641, while gene expression study showed that a group of genes are also deregulated by miR-7641, including many ribosomal proteins that are frequently co-expressed with *RPS16* in breast cancer. Direct inhibition of miR-7641 using a locked nucleic acid upregulated the expression of its target genes, sensitized cancer cells, and enhanced the efficiency of therapeutic agents such as doxorubicin. In addition, inhibition of miR-7641 boosted doxorubicin-mediated apoptosis of cancer cells via upregulation of apoptotic molecules Caspase 9 (CAS9) and poly ADP ribose polymerase (PARP) and downregulation of anti-apoptotic molecule BCL2. Thus, miR-7641 **might be** a clinically important cancer biomarker. Inhibition of miR-7641 expression could be an efficient treatment strategy for clinical patients.

## Introduction

MicroRNAs (miRNAs) are non-protein-coding RNA molecules that are thought to be involved in post-transcriptional regulation of approximately one third of human genes, either by inducing mRNA degradation or by inhibiting translation^1^. Under normal physiological conditions, the involvement of miRNAs is widespread, from sex differentiation and embryonic development, to cell proliferation, differentiation, and apoptosis^2, 3^. Deregulation of miRNA expression could accelerate disease progression and biological disorders, ranging from myocardial infarction and autoimmune diseases to tumorigenesis^4, 5^.

The involvement of miRNAs in cancer has been reported widely. Many miRNAs (such as miR-221, miR-222, miR-21, and miR-155) are regarded as oncogenic^6^, and overexpression of oncogenic miRNAs could enhance the proliferation, growth, and metastasis of cancers, and are considered as important biomarkers for the clinical diagnosis of cancers. In addition, suppression of these oncogenic miRNAs might improve therapeutic efficacy and increase the survival of patients^6, 7^. In contrast, there are many anti-cancer miRNAs (such as let-7, miR-26, miR-145, miR-23, miR-15, miR-16, miR-34a, miR-224, miR-143, and miR-921)^6, 7^, which are involved in the suppression and inhibition of cancers and could be used therapeutically.

However, the involvement of many miRNAs in cancer is poorly understood and not well-documented. In a previous study, it has been reported that mesenchymal stem cells (MSCs) derived exosomes contains many miRNAs, and some of them are poorly investigated, especially their roles in cancer are not evaluated^7^. The influential as well as regulatory interaction between MSCs and cancer is quite proven, while miRNAs might be a key tool that could mediate the interaction process^7^. In the present study, we selected top 10 miRNAs from the reported poorly investigated exosomal-miRNAs and evaluated their expression level in four cancers cell lines (two breast and two colon), as well as their response to doxorubicin treatment. Among them, miR-7641 showed very high expression in all cell lines, and was downregulated upon treating the cells with doxorubicin. Furthermore, inhibition of miR-7641 decreased cell viability and enhanced apoptotic-signaling molecules in different cancer cell lines. Additionally, the target genes of miR-7641 are connected with many other genes that are involved in breast and colorectal cancers, and alterations in those genes correlate with decreasing survival of cancer patients. Thus, miR-7641 could be an oncogenic miRNA and an important biomarker for the diagnosis of breast and colorectal cancers. Inhibition of miR-7641 could enhance the efficiency of cancer therapy by sensitizing cancer cells.

## Results

### MicroRNA-7641 and miR-1246 showed very high expression in different cancer cells

**Figure 1:**
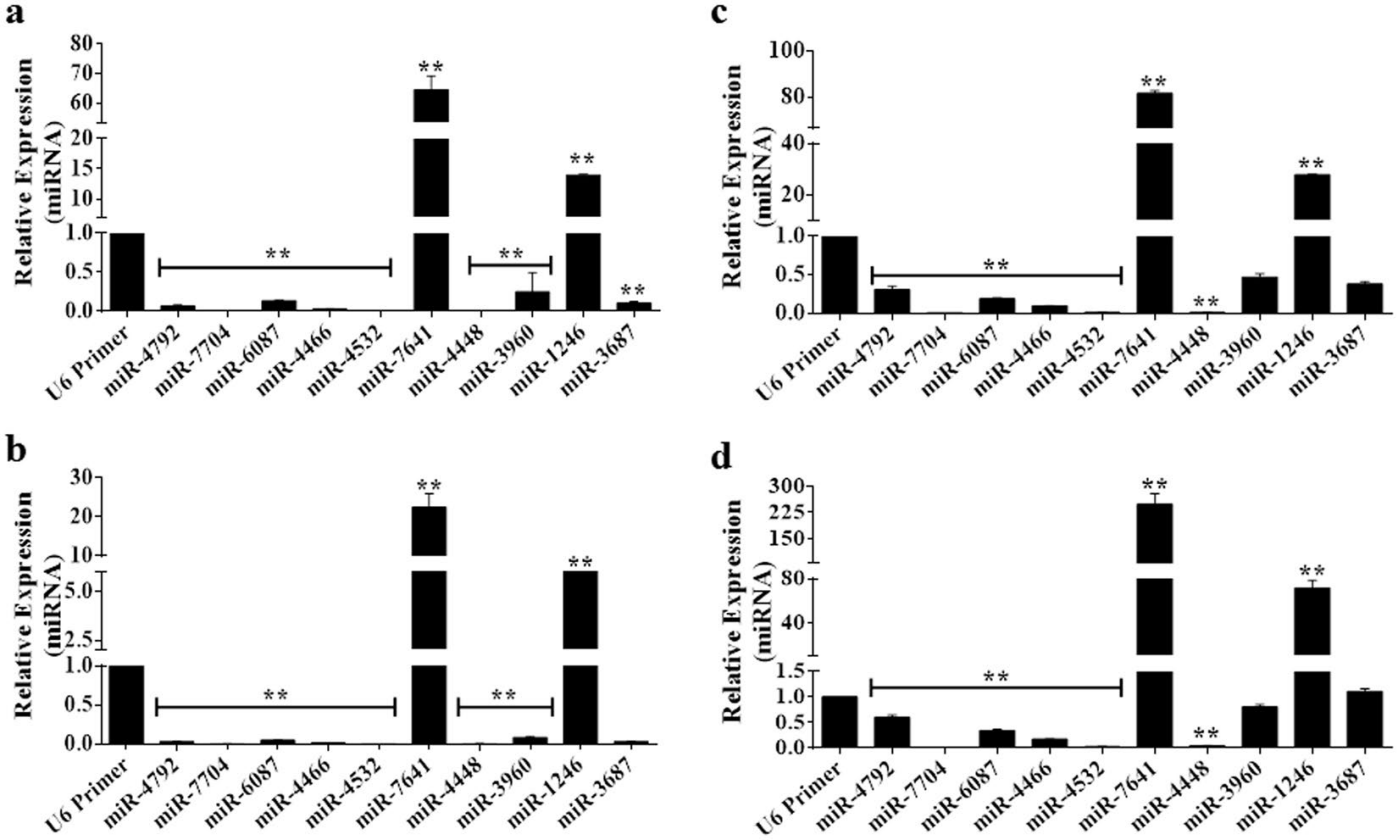
Expressions of 10 poorly investigated miRNAs in four cancer cell lines. (**a**) Bar diagram showing the expression levels of selected miRNAs in MCF-7 breast cancer cell line; the expression of miR-7641 was the highest followed by miR-1246, and the expression of both miRNAs were significantly (*p* < 0.01) higher than the U6 control expression. (**b**) Bar chart showing the expression levels of the 10 poorly investigated miRNAs in MDA-MB-231 breast cancer cells; miR-7641 and miR-1246 had significantly (*p* < 0.01) higher expression compared with the U6 control. (**c**) As shown in bar diagram, miR-7641 and miR-1246 were the highest expressed miRNAs in HT-29 colon cancer cells and their expressions were significantly (*p* < 0.01) higher than that of the U6 control. (**d**) Bar diagram showing the expression of 10 miRNAs in HCT116 colon cancer cells; miR-7641 and miR-1246 were detected as the highest expressed miRNAs and were expressed significantly (*p* < 0.01) higher than the U6 control. Bar diagrams presenting the mean ± SD obtained from triplicate experiments. *p < 0.05, **p < 0.01, and ***p < 0.001.

In this study, we evaluated the expression of 10 poorly studied miRNAs in four cancer cell lines, two breast cancer cell lines (MCF-7 and MDA-MB-231) and two colon cancer cell lines (HT-29 and HCT116). As shown in Fig. 1, eight miRNAs (miR-4792, miR-7704, miR-6087, miR-4466, miR-4532, miR-4448, miR-3960, and miR-3687) showed lower expression than the U6 control in all cancer cell lines. The remaining two miRNAs (miR-7641 and miR-1246) demonstrated significantly (p < 0.01) higher expression (up to several hundred folds greater than the U6 control) in all cancer cell lines (Fig. 1). Thus, miR-7641 and miR-1246 might have important roles in carcinogenesis, and silencing the expression of these miRNAs could sensitize cancer cells, in addition to enhancing the efficacy of therapeutic treatments.

### Doxorubicin downregulated the expression of miR-7641 and miR-3960

**Figure 2:**
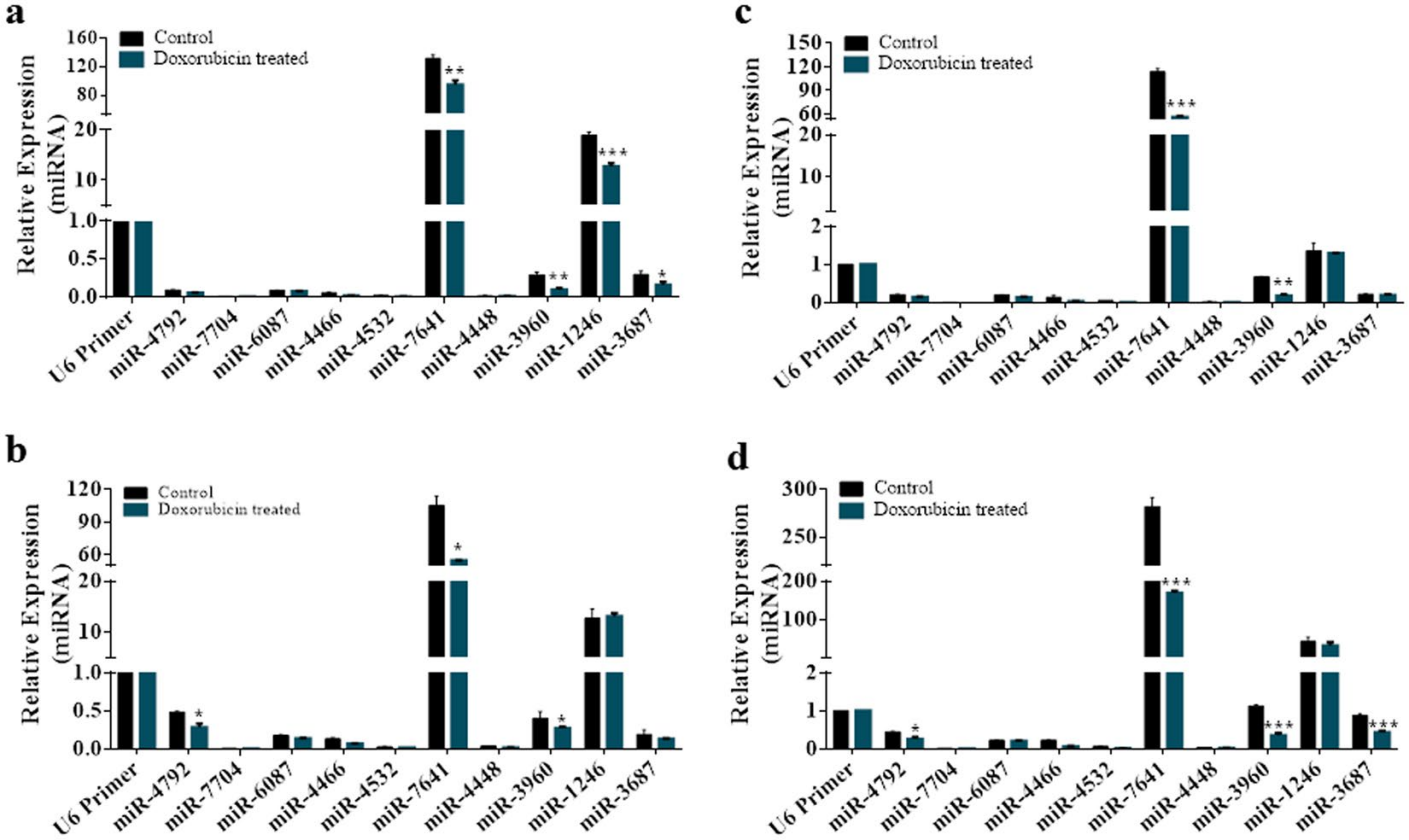
Doxorubicin-mediated regulation of miRNA expression in cancer cells. (**a**) Bar diagram presenting miRNA expression in doxorubicin treated and untreated MCF-7 breast cancer cells. Significant downregulation was observed for several miRNAs upon doxorubicin treatment, such as miR-7641 (*p* < 0.01), miR-3960 (*p* < 0.01), miR-1246 (*p* < 0.001), and miR-3687 (*p* < 0.05). (**b**) As shown in the bar diagram, significant downregulation of miR-4792, miR-7641, and miR-3960 (*p* < 0.05) was observed in doxorubicin treated MDA-MB-231 breast cancer cells compared with untreated control cells. (**c**) Bar diagram showing that doxorubicin treatment resulted in significant downregulation of miR-7641 and miR-3960 expression (*p* < 0.001, *p* < 0.01, respectively) in HT-29 colon cancer cells. (**d**) As shown in bar chart, doxorubicin treatment downregulated the expressions of several miRNAs, including miR-4792 (*p* < 0.05), miR-7641 (*p* < 0.001), miR-3960 (*p* < 0.001), and miR-3687 (*p* < 0.001) in HCT116 colon cancer cells. Bar diagram shows the mean ± SD obtained from triplicate experiments. *p < 0.05, **p < 0.01, and ***p < 0.001.

The regulatory effect of doxorubicin on the 10 selected miRNAs was determined by comparing the expression level of the miRNAs in doxorubicin treated cancer cells and non-treated control cells. As shown in Fig. 2, doxorubicin treatment resulted in significant (p < 0.05/p < 0.01/p < 0.001) downregulation of miR-7641 and miR-3960 in all four cell lines. In addition, miR-3687 expression declined significantly in doxorubicin treated MCF-7 cells (Fig. 2a) and HCT116 (Fig. 2d) cells (p < 0.05 and p < 0.001, respectively). As well, miR-1246 and miR-4792 expression decreased in doxorubicin treated MCF-7 cells (Fig. 2a) and MDA-MB-231 breast cancer cells (Fig. 2c), respectively. Hence, several of the selected miRNAs might be influenced with doxorubicin treatment. In particular, miR-7641 was downregulated in all cancer cell lines upon doxorubicin treatment and could play an important role during doxorubicin-mediated killing of cancer cells.

### A miR-7641-inhibitor enhances the efficacy of doxorubicin

**Figure 3:**
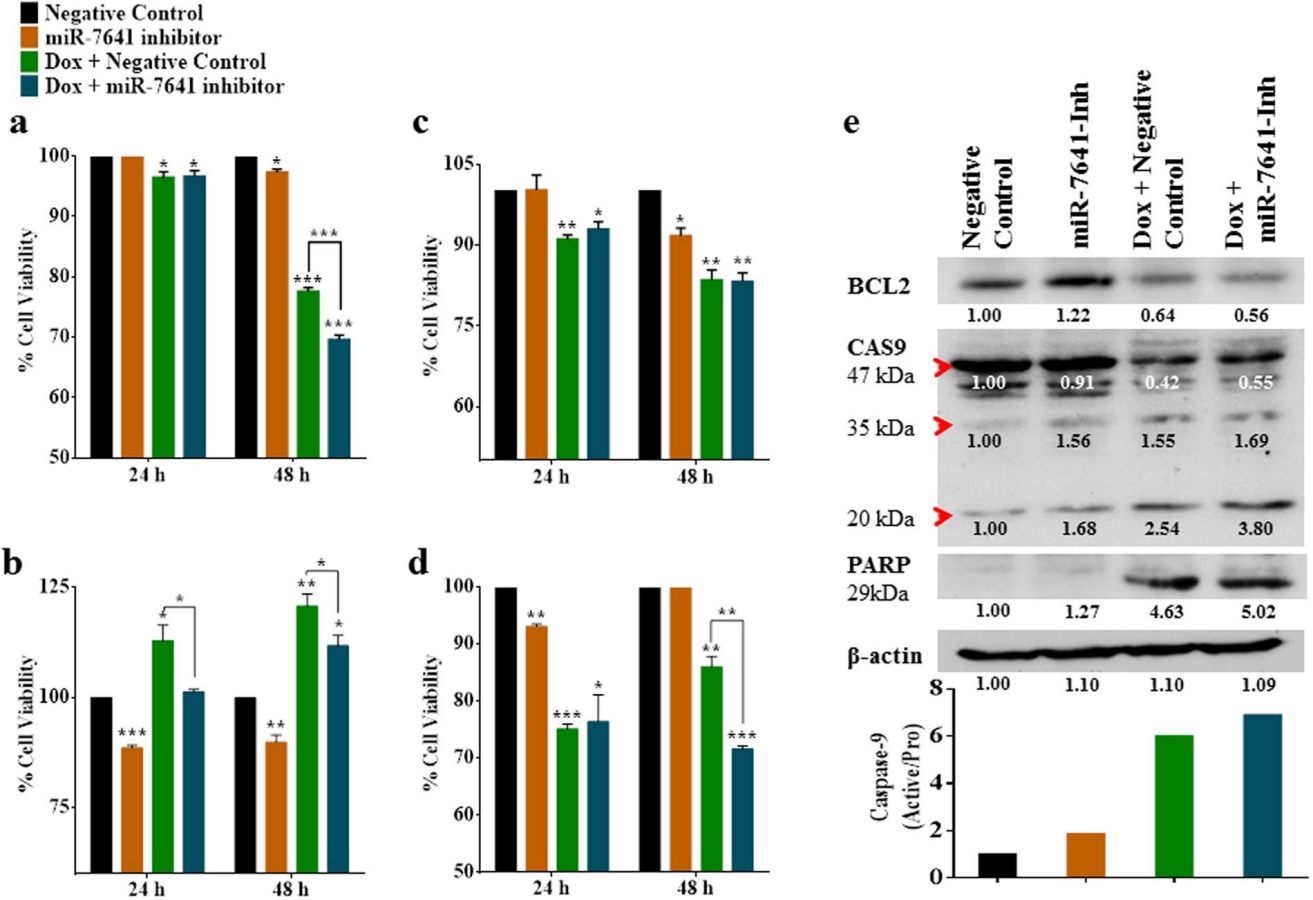
Inhibition of miR-7641 reduced the viability of cancer cells and enhanced the efficiency of doxorubicin treatment. (**a**) As shown in bar diagram, inhibition of miR-7641 reduced the viability of MCF-7 breast cancer cells, and a treatment strategy using both doxorubicin and the miR-7641-inhibitor was significantly (*p* < 0.001) more effective than doxorubicin plus the negative control treatment. (**b**) Bar diagram showing that the miR-7641-inhibitor could sensitize MDA-MB-231 breast cancer cells. Treatment with both doxorubicin and the miR-7641-inhibitor reduced cell viability significantly (*p* < 0.05) compared with the doxorubicin plus negative control group. (**c**) As shown in the bar diagram, the miR-7641-inhibitor reduced viability of HT-29 colon cancer cells significantly (*p* < 0.05) at 48 h post-transfection. However, there was no significant difference between the doxorubicin plus miR-7641-inhibitor group and the doxorubicin plus negative control group. (**d**) Bar diagram showing that the miR-7641-inhibitor could sensitize HCT116 colon cancer cells, and that doxorubicin plus miR-7641-inhibitor treatment was significantly (*p* < 0.01) more effective than doxorubicin and negative control treatment. (**e**) Western blotting analysis showing that doxorubicin plus the miR-7641-inhibitor treatment was more effective in enhancing the upregulation of apoptotic molecules, such as CAS9 and PARP, and downregulation of anti-apoptotic molecule BCL2 in MCF-7 breast cancer cells compared with the doxorubicin and negative control treated group. Bar diagrams show the mean ± SD obtained from triplicate experiments. *p < 0.05, **p < 0.01, and ***p < 0.001.

The experimental dose of doxorubicin (0.35 µg/mL) and treatment time (48 h) were selected based on a dose- and time-dependent treatment of cancer cells using a series of doxorubicin concentrations (0, 0.1, 0.2, 0.3, 0.4, 0.5, 0.6, 0.7, 0.8, 0.9, and 1.0 µg/mL) for 24 h and 48 h (Supplementary Fig. S1). As shown in Supplementary Figure S2, the effectiveness of the miR-7641 inhibitor was measured by detecting the expression level of miR-7641 at 24, 48, and 72 h post-transfection: 48 h was observed to be the most suitable time. Thus, the cancer cell lines were transfected with 30 nM miR-7641-inhibitor or a negative control miRNA for 6 h and then incubated for around 48 h with or without 0.35 µg/mL doxorubicin. As shown in Fig. 3, compared with the negative control (miRNA control), the miR-7641-inhibitor alone reduced the viability of cancer cells significantly (p < 0.05/p < 0.01/p < 0.001) in most cases. Similarly, combination treatment using both the miR-7641-inhibitor and doxorubicin was much more effective than doxorubicin plus the negative control (miRNA control), and differed significantly in MCF-7 cells (p < 0.001), MDA-MB-231 cells (p < 0.05), and HCT116 cells (p < 0.01).

Moreover, the regulation of apoptosis related molecules, including BCL2, CAS9, and PARP, were detected in MCF-7 cells under different treatment conditions. The results showed lower levels of the anti-apoptotic molecule BCL2 in miR-7641-inhibitor plus doxorubicin treated cells compared with that in the doxorubicin plus negative control (miRNA control) treated cells (Fig. 3e). By contrast, the miR-7641-inhibitor plus doxorubicin treatment upregulated pro-apoptotic molecules (CAS9 and PARP) compared with the doxorubicin plus negative control (miRNA control) treatment (Fig. 3e). These data suggested that targeting miR-7641 could enhance the efficacy of cancer therapy.

### MiR-7641 target genes belong to numerous biological and functional categories

Around 3500 genes were predicted to be targets of miR-7641 (Supplementary Spreadsheet S1), however, most of the genes have low context score, indicating low binding probability of the miR-7641 to the predicted target site. In addition, there are also some nonfunctional pseudogenes among the predicted targets. The functional target genes were also predicted to be involved in numerous biological, physiological, and metabolic processes and belong to different protein classes. As shown in Supplementary Figure S3a, gene ontology (GO) molecular function analysis suggested that around 40% of the target genes are binding molecules; 36% are involved in catalytic activity; and transport activity and receptor activity encompassed around 7% of the molecules each. The miR-7641 target list also included molecules that are involved in signal transduction, structural components, translation regulation, channel regulation, and antioxidant activity. As shown in Supplementary Figure S3b, GO analysis for cellular components identified that around 40% encompassed cell part, 24% organelles, 15% membrane, 13% macromolecular complex, and 5% extracellular region. Mir-7641 target genes also represented numerous protein classes, such as nucleic acid binding proteins (14.74%), hydrolases (9.57%), transcription factors (9.54%), enzyme modulators (8.13%), transferases (7.82%), receptors (7.20%), signaling molecule (6.13%), transporters (6.09%), and many others (Supplementary Fig. S3c). The GO biological processes analysis is presented in Supplementary Figure S3d, in which most of the molecules are involved in cellular and metabolic processes, which comprise 30% and 25%, respectively. Molecules are also involved in biogenesis, localization, and biological regulation, responses to stimuli, developmental processes, immune system processes, and multicellular organismal processes (Supplementary Fig. S3d). Therefore, miR-7641 has the potential to interfere with numerous physiological and biological processes.

### Inhibition of miR-7641 upregulated several of the targets genes in different cancer cell lines

**Figure 4:**
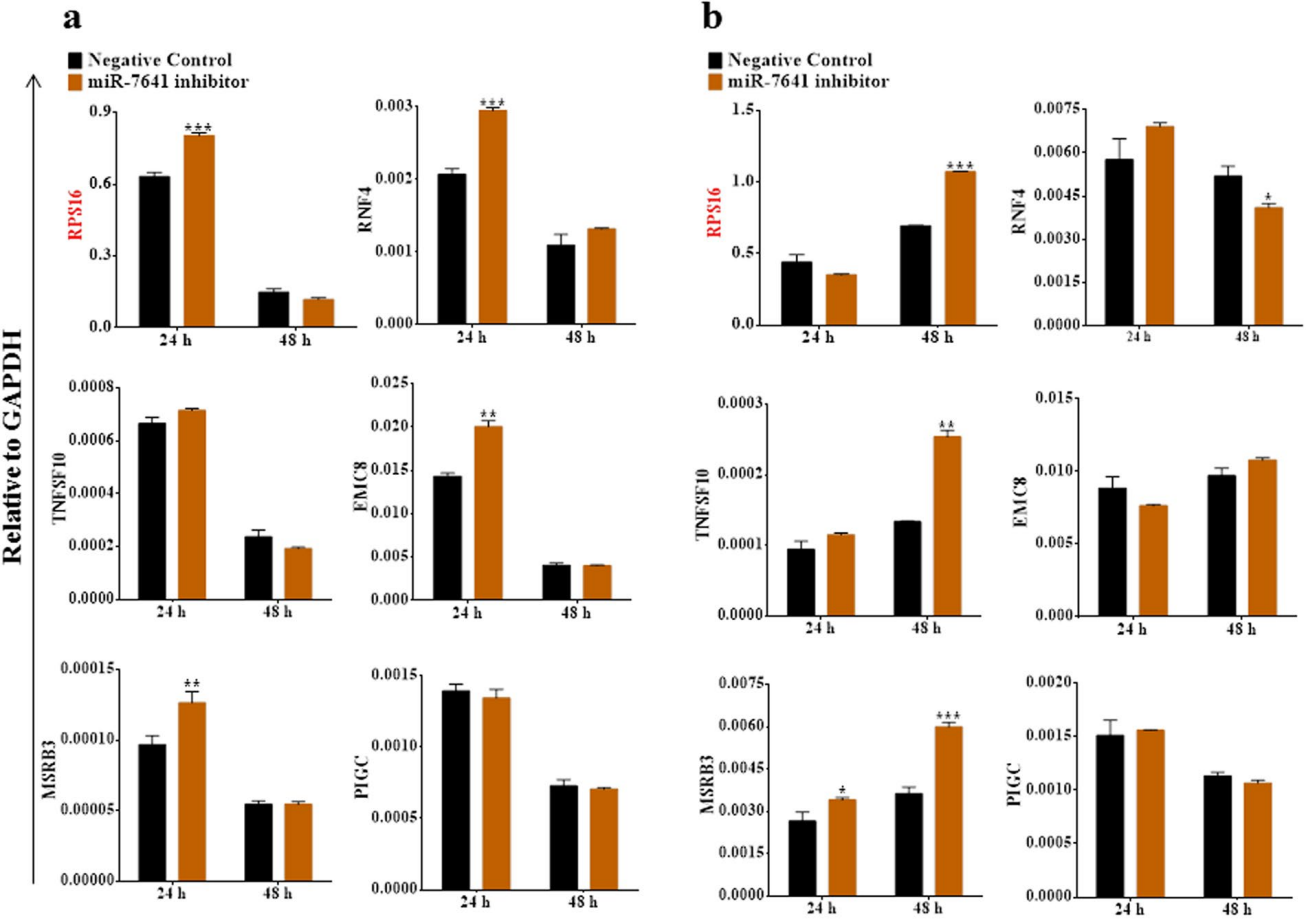
Inhibition of miR-7641 altered target gene expression in breast cancer cells. (**a**) Bar diagrams showing that inhibition of miR-7641 upregulated the expressions of *RPS16*, *RNF4*, *EMC8*, and *MSRB3* in MCF-7 breast cancer cells. (**b**) As shown in the bar diagrams, inhibition of miR-7641 upregulated the expressions of *RPS16*, *TNFSF10*, and *MSRB3* in MDA-MB-231 breast cancer cells. Bar diagrams show the mean ± SD obtained from triplicate experiments. *p < 0.05, **p < 0.01, and ***p < 0.001.

Initially, 12 target genes of miR-7641 were selected for validation, the detail about miR-7641 binding site to the respective 12 genes are given in Supplementary Table S1. MirVana™ miRNA inhibitor for miRNA-7641 was used to suppress the expression of miR-7641. Upon inhibition of miR-7641 expression, the expressions of several of the target genes (*RPS16*, *RNF4*, *EMC8*, and *MSRB3*) increased, *CUL3* expression **de**creased, and other genes, including *PIGC*, remained unchanged in MCF-7 breast cancer cells (Fig. 4a and Supplementary Fig. S4a). Similarly, miR-7641 inhibition in MDA-MB-231 breast cancer cells upregulated *RPS16*, *TNFSF10*, *MSRB3*, *CDNF*, *ZNF616*, and *NBEA*, downregulated *CUL3*, and showed no remarkable changes for *PIGC* and the other genes (Fig. 4b and Supplementary Fig. S4b).

**Figure 5:**
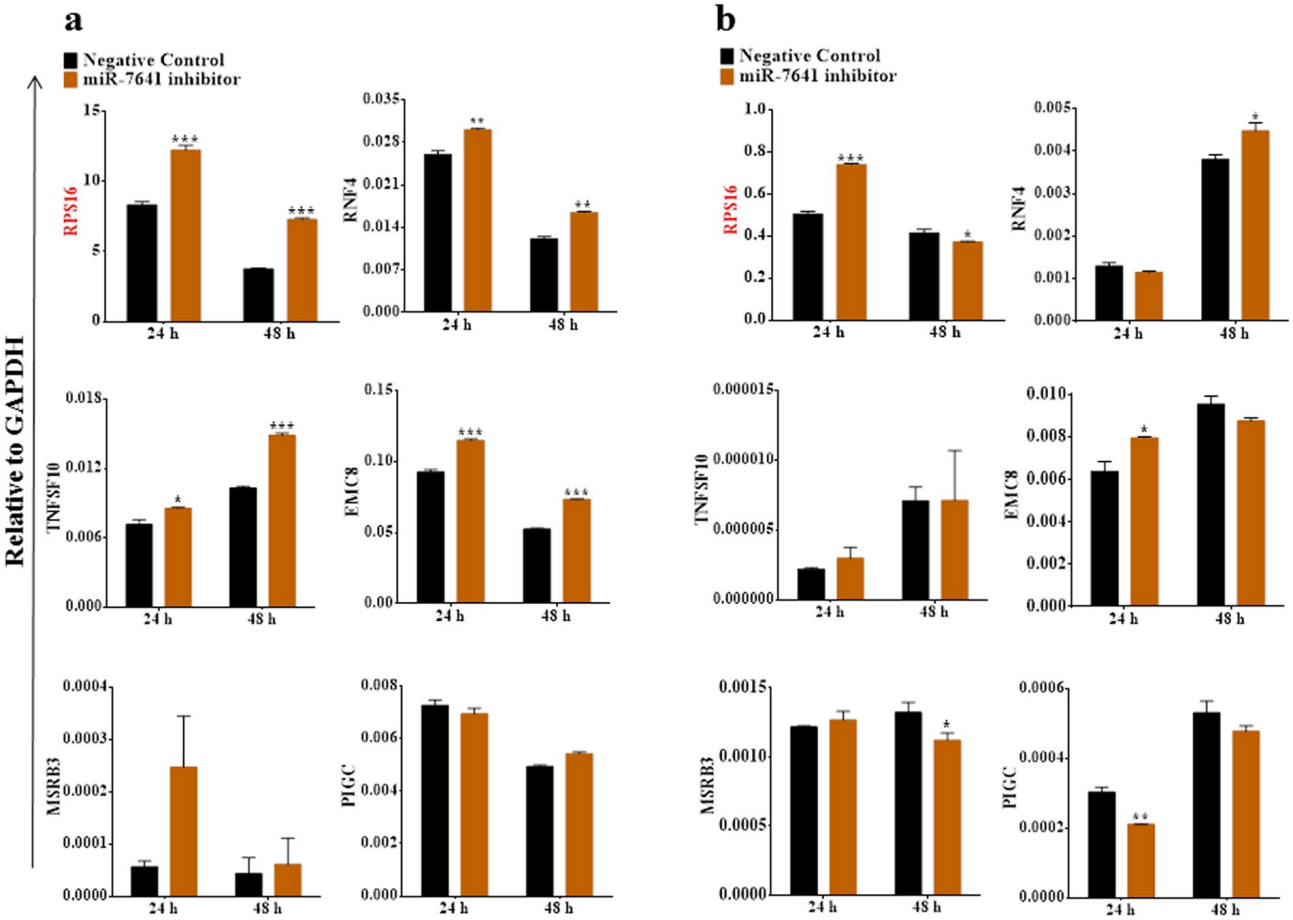
Inhibition of miR-7641 altered target gene expression in colon cancer cells. (**a**) Bar diagrams presenting the expression patterns of miR-7641 target genes in HT-29 colon cancer cells upon transfection with a locked nucleic acid inhibitor against miR-7641. Inhibition of miR-7641 resulted in the upregulation of *RPS16*, *RNF4*, *TNFSF10*, and *EMC8* expression. (**b**) Bar diagrams showing that inhibition of miR-7641 upregulated *RPS16*, *RNF4*, and *EMC8* expression in HCT116 colon cancer cells. Bar diagrams show the mean ± SD obtained from triplicate experiments. *p < 0.05, **p < 0.01, and ***p < 0.001.

As shown in Fig. 5a and Supplementary Figure S5a, in the HT-29 cell line, inhibition of miR-7641 caused upregulation of the *RPS16*, *RNF4*, *TNFSF10*, *EMC8*, *MSRB3*, *CUL3*, *CDNF*, and *ZNF616* genes, downregulation of *SIGLEC7*, and no change for other genes including *PIGC*. In HCT116 cells, *RPS16*, *RNF4*, *CUL3*, and *ZNF616* were upregulated by the inhibition of miR-7641 (Fig. 5b and Supplementary Fig. S5b). These findings validated the predicted targets of miR-7641 in different cell lines and supported the possible involvement of miR-7641 in cancers, although the targeting efficiency could vary depending on the cell line; however, several genes, such as *RPS16*, *RNF4*, *TNFSF10*, *EMC8*, and *MSRB3* were downregulated in most of the cell lines and *PIGC* remained unchanged in all cell lines. Thus, the *RPS16*, *RNF4*, *TNFSF10*, *EMC8*, and *MSRB3* genes could be the real targets of miR-7641. Especially, *RPS16* responded to the inhibition of miR-7641 in all cells lines, indicating that *RPS16* is a universal target of miR-7641.

### Luciferase reporter assay confirmed that RPS16 and TNFSF10 are real targets of miR-7641

**Figure 6:**
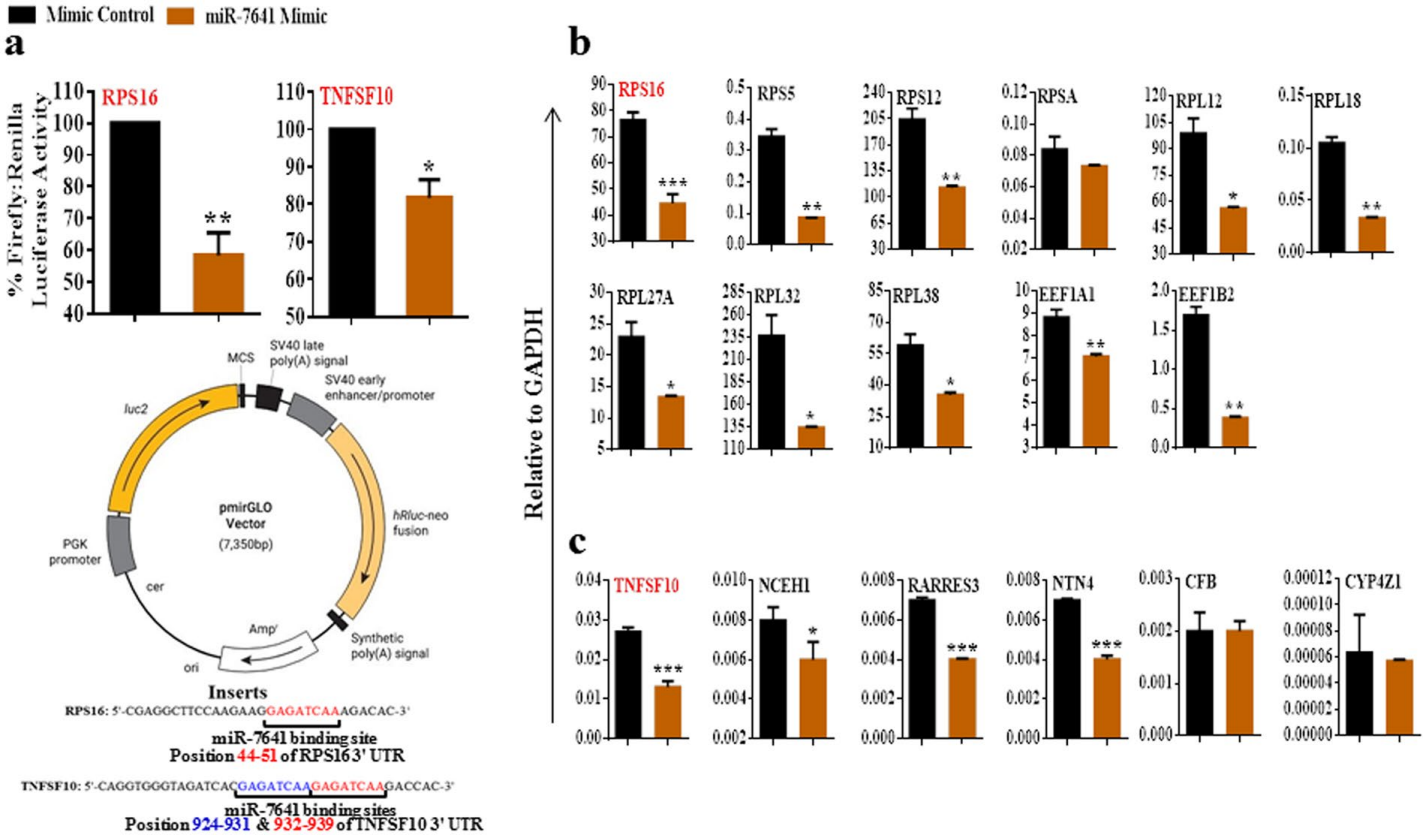
*Luc gene* activity assay confirmed *RPS16* and *TNFSF10* as target genes of miR-7641. (**a**) In both cases, bar diagrams showed that the ratio of firefly and renilla luciferase activity was significantly lower (*p* < 0.01/*p* < 0.05) in cells co-transfected with miR-7641 compared to the mimic negative control co-transfected cells. The pmiRGLO vector was used for the expression of miR-7641 target site, while the insert oligo sequence showing miR-7641 binding site in both *RPS16* (44–51, of *RPS16* 3′ UTR) and *TNFSF10* (924–931 & 932–939, of *TNFSF10* 3′ UTR) genes. (**b**) Bar diagrams showing the reduced expression of *RPS16* and 10 other genes (*RPS5*, *RPS12*, *RPSA*, *RPL12*, *RPL18*, *RPL27A*, *RPL32*, *RPL38*, *EEF1A1* and *EEF1B2*) that are frequently co-expressed in breast cancer patients, in MCF-7 breast cancer cells upon transfection of miR-7641 compared to the mimic negative control transfected cells. (**c**) Bar diagrams presenting the expression of *TNFSF10* along with five other genes (*NCEH1*, *RARRES3*, *NTN4*, *CFB* and *CYP4Z1*) that are frequently co-expressed in breast cancer patients, in miR-7641 transfected MCF-7 cells compared to the mimic negative control transfected cells. *TNFSF10*, *NCEH1*, *RARRES3* and *NTN4* were significantly reduced upon miR-7641 transfection, while *CFB* and *CYP4Z1* showed no significant changes in expression. Bar diagrams show the mean ± SD obtained from triplicate experiments. *p < 0.05, **p < 0.01, and ***p < 0.001.

As shown in Fig. 6a, *Luc gene* expression vector containing miR-7641 binding site for either *RPS16* or *TNFSF10* co-transfected with miR-7641 mimic showed significant reduction of luciferase activity compared to the group co-transfected with ‘mir Vana™ miRNA Mimic negative Control’. Reduction of luciferase activity was around 50% in case of binding site from *RPS16* (p < 0.01), while it was around 20% in case of binding site from *TNFSF10* (p < 0.05). The detail data related to luciferase activity assay is provided in the Supplementary Table S2. These data indicating that miR-7641 could successfully bind to its targeting site for both *RPS16* and *TNFSF10*, thus, both *RPS16* and *TNFSF10* are proven target genes of miR-7641. However, the targeting efficacy for *RPS16* was much higher than the targeting efficacy for *TNFSF10*.

### miR-7641 showed regulatory influence on genes that frequently co-expressed with RPS16 or TNFSF10

As shown in Fig. 6b, expression of top 10 genes (*RPS5*, *RPS12*, *RPSA*, *RPL12*, *RPL18*, *RPL27A*, *RPL32*, *RPL38*, *EEF1A1 and EEF1B2*) that are frequently co-expressed with *RPS16* in breast cancer patients, have been evaluated upon transfection with miR-7641 mimic. The list of genes that frequently co-express with *RPS16* in breast cancer patients are enlisted in Supplementary Table S3. Nine out of 10 selected genes showed significant (p < 0.05/p < 0.01/p < 0.001) downregulation in cells transfected with miR-7641 mimic compared to the ‘mir Vana™ miRNA Mimic negative Control’ transfected cells. These data indicated that miR-7641 have regulatory effect on *RPS16*, as well as to the genes that are mutually co-expressed with *RPS16* in breast cancer. Interestingly, these genes are mainly ribosomal proteins; therefore, miR-7641 could play important role during the regulation of ribosomal proteins.

Moreover, expression of top five genes (*NCEH1*, *RARRES3*, *NTN4*, *CFB and CYP4Z1*) that are frequently co-expressed with *TNFSF10* in breast cancer patients, have been detected upon transfection with miR-7641 mimic (Fig. 6c). All genes that frequently co-express with *TNFSF10* in breast cancer patients are enlisted in Supplementary Table S4. Data showed that miR-7641 mimic significantly downregulated the expression of *NCEH1*, *RARRES3* and *NTN4* along with *TNFSF10*, while *CFB* and *CYP4Z1* did not show significant variation compared to the ‘mir Vana™ miRNA Mimic negative Control’ transfected cells. These results indicated that miR-7641 could also influence the expression of genes that are mutually co-expressed with *TNFSF10* in breast cancer, at least in partial.

### Alteration of RPS16 and TNFSF10 along with the mutually co-expressed genes correlates to patient’s survival

**Figure 7:**
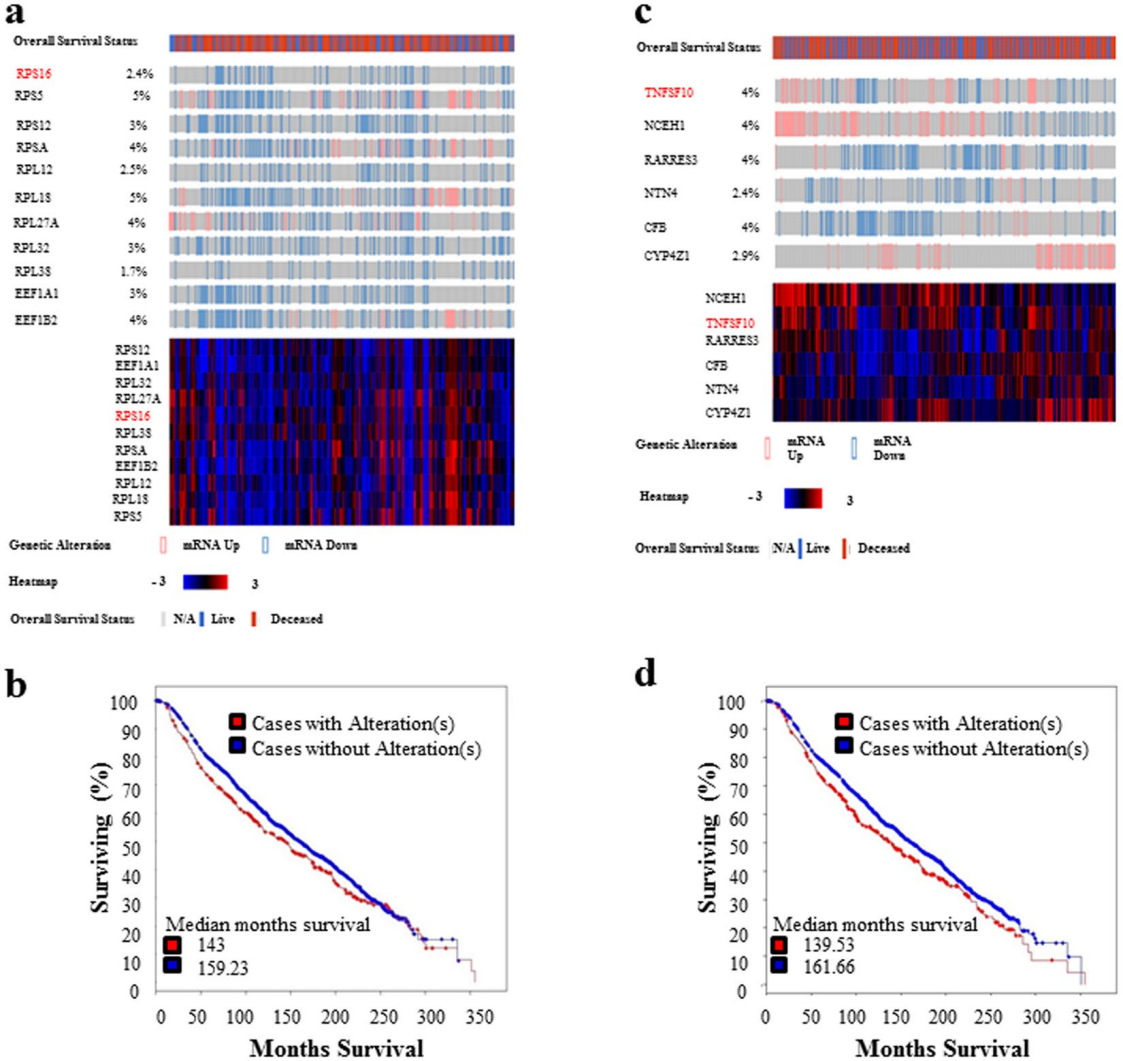
Alternations of *RPS16* and *TNFSF10* along with their mutually co-expressed genes might correlate the survival status in breast cancer patients. (**a**) Oncoprint of *RPS16* and its mutually co-expressed 10 genes (*RPS5*, *RPS12*, *RPSA*, *RPL12*, *RPL18*, *RPL27A*, *RPL32*, *RPL38*, *EEF1A1* and *EEF1B2*) in breast cancer patients, overall survival status of the patients is shown at the top bar, while heatmap (bottom) presenting the overall expression profile of the genes. (**b**) Survival graph showing that alterations in the expression of *RPS16* and its mutually co-expressed 10 genes are negatively correlated with the survival status of breast cancer patients. (**c**) Oncoprint of *TNFSF10* and its five mutually co-expressed genes (*NCEH1*, *RARRES3*, *NTN4*, *CFB* and *CYP4Z1*) in clinical breast cancer patients. Overall survival status of the patients is shown at the top, while heatmap at the bottom presenting the overall expression profile of the genes. (**d**) Survival graph showing that alterations of *TNFSF10, NCEH1*, *RARRES3*, *NTN4*, *CFB* and *CYP4Z1* are negatively correlated with the overall survival status of breast cancer patients. The data were generated by the TCGA Research Network: http://cancergenome.nih.gov/.”

Figure 7a showed the alteration status of *RPS16* and the top 10 genes that are frequently co-expressed with *RPS16* in the breast cancer patients. As shown in Fig. 7a, most of the cases the genes have been downregulated, without few exceptions where some of the genes become upregulated. Moreover, the alteration of *RPS16* along with the 10 mutually expressed genes including *RPS5*, *RPS12*, *RPSA*, *RPL12*, *RPL18*, *RPL27A*, *RPL32*, *RPL38*, *EEF1A1* and *EEF1B2* showed negative correlation with patient’s overall survival status. In brief, the overall month’s survival has been decreased in case of patients having altered expression for the respective genes (143 months) compared to the patients without alteration (159.23 months) (Fig. 7b). Furthermore, *RPS16* along with the top 10 mutually co-expressed genes also showed intensive connectivity with many other genes, and most of them are ribosomal proteins (Supplementary Fig. S6). These data indicated that *RPS16* and its mutually co-expressed genes might have very important involvement in the breast cancer, while upregulated expression of miR-7641 might be one of the reasons behind the downregulated expression of ribosomal proteins in breast cancer patients.

As shown in Fig. 7c, alteration of *TNFSF10* and its mutually co-expressed genes are also investigated in clinical breast cancer patients, however, both upregulated and downregulated expression of those genes were detected. Furthermore, the alteration of these genes also correlates to patient’s survival. The overall month’s survival of the patients decreased in case of alteration of *TNFSF10* and its mutually co-expressed partner genes (139.53 months) compared to the patients without alteration of the concerned genes (161.66 months) (Fig. 7d). Thus, these genes also might have influential involvement in breast cancers.

## Discussion

Not only protein coding genes, but also non-coding genes, including miRNAs, are important regulators of biological and physiological processes^3^, and deregulation of miRNA expression could accelerate the progression of many diseases, such as myocardial infarction, autoimmune disease, viral diseases, neurodegenerative diseases, and cancers^4, 5, 8^. Both oncogenic and anti-oncogenic miRNAs have been identified to play active roles in different cancers^6^. In addition, a particular miRNA could be oncogenic for one type of cancer, as well as anti-oncogenic for other types^7^. Hence, investigating the roles of miRNAs is required to understand the mechanisms and treatment strategies of diseases.

Despite their importance, the regulatory properties and functions of many miRNAs are unknown and deserved to be explored^7^. The data presented here showed that miR-7641, which was a hitherto poorly investigated miRNA, is expressed dominantly in different breast and colon cancer cell lines. Inhibition of miR-7641 reduced the proliferation of cancer cells and enhanced the efficiency of doxorubicin. In addition, altered expression of the target genes of miR-7641 correlated negatively with survival of clinical cancer patients. In this study, we showed that two (miR-7641 and miR-1246) out of 10 poorly investigated miRNAs had extraordinarily high expression in all four cancer cell lines compared with the U6 control expression, indicating that miR-7641 and miR-1246 might have oncogenic properties. The oncogenic properties of miR-1246 have already been demonstrated in lung cancer^9, 10^, hepatocellular carcinoma^11^, colorectal cancer^12^, and others^13^. However, the only published article concerning miR-7641 described its role during endothelial differentiation from embryonic stem cells^14^; our study is the first report of miR-7641 as a regulator of ribosomal proteins and a promising targeting factor for cancer therapy.

Accordingly, we have shown that upon treatment with doxorubicin, miR-7641 was downregulated in the four cancer cell lines, suggesting that doxorubicin cooperates with miR-7641 during the regulation of anti-cancer signaling. These findings also signified the involvement of miR-7641 in cancers and the potential of miR-7641 as a therapeutic target in cancer therapy. Furthermore, a miR-7641-inhibitor reduced the proliferation of all cancer cell lines, and the combination treatment of the miR-7641 inhibitor and doxorubicin decreased cell viability and upregulated apoptotic molecules (CAS9, PARP) much more effectively in different cancer cell lines compared with doxorubicin alone. Considering these findings, it is clear that inhibition of miR-7641 could sensitize cancer cells and enhance the efficiency of therapeutic agents.

*In silico* analysis using the Panther database showed that the predicted target genes of miR-7641 are a dynamic category and have roles in numerous molecular, cellular, and biological processes. Among the targets, the 12 top-scoring genes were considered for validation to confirm the real targets of miR-7641. The data showed that inhibition of miR-7641 upregulated *RPS16* in all cancer cell lines, while *TNFSF10*, *RNF4*, *EMC8* and *CUL3* responded irregularly. These data indicated that *RPS16* might be a universal target gene of miR-7641, while other might be dependent on the cell line and niche. Further, the luciferase activity assay confirmed that *RPS16* and *TNFSF10* are two real targets of miR-7641; however, the binding efficacy of miR-7641 to *RPS16* is much higher compared to the binding efficacy to *TNFSF10*. Altogether, *RPS16* might be the most authentic and universal target gene of miR-7641.

Our results also showed that nine out of the 10 genes that are frequently co-expressed with *RPS16* in breast cancer patients are also significantly downregulated upon transfection of the cells with miR-7641 mimic, confirming that miR-7641 can really target for *RPS16*, as well as it could regulate the expression of a panel of ribosomal proteins, indirectly. Hence, miR-7641 might be a very important miRNA that might modulate ribosomal protein translation and expression processes in breast cancer patients. In addition, these genes are members of exclusive networks with many other genes involved in breast cancers. Thus, alteration in the expressions of these genes could modify the expressions of other genes in the same network, ultimately affecting the fate of cancer patients. Moreover, there was a correlation between these ribosomal proteins expression and survival of breast cancer patients: changes in one or more of the target gene(s) could reduce the median survival of the cancer patients. Hence, overexpression of miR-7641 might reduce the survival of cancer patients via modification of the ribosomal proteins panel, while inhibition of miR-7641 could be an effective strategy to readjust the expressions of these genes to normal levels, which might enhance the survival of cancer patients.

In conclusion, miR-7641 could be recognized as an oncogenic miRNA that might be involved in the alteration of the ribosomal proteins expressions, as well as triggering the oncogenic process. At least, the present study demonstrated the involvement of miR-7641 in the regulation of ribosomal proteins, as well as its potential regulatory influence on the breast and colorectal cancers. Moreover, overexpression of miR-7641 could be used as a biomarker for the diagnosis of breast and colorectal cancer patients. In addition, locked nucleic acid inhibitors targeting miR-7641 could be used in combination with other therapeutic agents, such as doxorubicin, to enhance the efficiency of cancer therapy.

## Material and Methods

### Kits and Reagents

Reagent and kits were derived from different commercial companies, such as a Small-RNA isolation kit (RA808A-1; SBI, Palo Alto, CA, USA), Mir-X™ miRNA First Strand Synthesis and SYBR® qRT-PCR kit (Cat. No. 638316; Clontech Laboratories, Inc., Mountain View, CA, USA), Dual-Luciferase® Reporter Assay System (Cat. No. E1910; Promega Corporation, Madison, WI, USA) and Dulbecco’s modified Eagle’s medium (DMEM)-high glucose medium and phosphate buffered saline (PBS; Hyclone Laboratories, Inc. UT, USA). Roswell Park Memorial Institute (RPMI) medium, penicillin-streptomycin (PS) solution, and trypsin-EDTA (TE) solution were obtained from Life Technologies GIBCO (Grand Island, NY, USA). Antigen probing primary antibodies used for immunoblotting against BCL2 (Santa Cruz, CA, USA), CASP9, PARP, and β-actin (Abcam, Cambridge, MA, USA). Fetal bovine serum (FBS) and other analytical grade reagents were purchased from Sigma-Aldrich (St. Louis, MO, USA), unless otherwise stated.

### Cell lines and culture

Two breast cancer cell lines (MCF-7 and MDA-MB-231) and two colon cancer cell lines (HT-29 and HCT116) were used to investigate the role of miR-7641 in cancer. MCF-7, HT-29, and HCT116 cell lines were maintained in DMEM-high glucose medium, while MDA-MB-231 cells were cultured in RPMI medium. Supplementation with 10% FBS and 100 U/mL PS was used to fortify the basal medium for all cell lines. The cells were incubated in a humidified incubator at 37 °C and 5% CO_2_; medium was replaced and the cells were fed with fresh medium at around every 48 to 72 h. Passaging and amplification of cells were performed during 90 to 100% confluency.

### Small-RNA isolation

RNA was isolated using the small-RNA isolation kit according to the manufacturer’s guidelines, with some modifications. Briefly, cells were harvested with 0.25% TE, pelleted at 500 × *g* and transferred to a 1.5-mL Eppendorf tube. Then, 350 µL of lysis buffer was added to 1 × 10^6^ cells, vortexed for 10 s, incubated for 5 min at room temperature (RT), transferred to QIAshredder spin column (Qiagen, Hilden, Germany) with a collection tube, and centrifuged at 16,000 × *g* for 2 min in a microfuge. The flow through was collected, supplemented with 200 µL of 100% ethanol, vortexed for 10 s, transferred to the miRNA binding spin column with a collection tube, and centrifuged at 16,000 × *g* for 1 min. The flow through was discarded; the spin column was washed twice with 400 µL of wash buffer, and then transferred to a new collection tube. Small-RNA was collected in 30-µL elution buffer and stored at −80 °C for downstream analysis.

### cDNA synthesis and miRNA detection

MiRNA expression was detected by quantitative real-time reverse transcription PCR (qRT-PCR) according to the guidelines provided with Mir-X miRNA qRT-PCR SYBR kit (Clontech Laboratories, Inc., CA, USA). In brief, a single-step polyadenylation and reverse-transcription reaction was performed to prepare cDNAs using the RNA samples derived from cancer cell lines. For qRT-PCR analysis, particular miRNA sequences were regarded as miRNA-specific 5′ primers, and the mRQ 3′ primers supplied with the kit were used as the 3′ primers for all miRNAs. The U6 RNA was amplified as a control. Primer sets are listed in Supplementary Table S5.

### Cell-cytotoxicity assay

The dose dependent effect of doxorubicin was evaluated by treating different cancer cell lines with a series of doses (0, 0.1, 0.2, 0.3, 0.4, 0.5, 0.6, 0.7, 0.8, 0.9, and 1.0 µg/mL). Cell viability was measured at 24 and 48 h post-treatment using a Cell Counting Kit-8 (CCK-8; Dojindo Laboratories, Kumamoto, Japan). The synergistic influence of a locked nucleic acid inhibitor against miR-7641 and doxorubicin was investigated by treating the cells with 0.35 µg/mL doxorubicin after transfection with 30 nM of the miR-7641-inhibitor for 6 h. The ‘mir Vana™ miRNA Mimic negative Control’ was considered as the miRNA control. Cell cytotoxicity was detected using CCK-8 assay system at 24 and 48 h post-treatment.

### Immunoblotting

Radio-immunoprecipitation lysis buffer was used to prepare protein lysates, and the concentration of protein was determined using a BCA protein assay kit (Thermo Scientific). Proteins were separated using 12% SDS-PAGE and transferred electrophoretically to polyvinylidene fluoride membranes. The membranes were incubated with 5% skim milk for 2 h at RT, overnight at 4 °C with primary antibodies, and for 1 h with horseradish peroxidase-conjugated secondary antibody at RT. Immuno-reactivity was detected using enhanced chemiluminescence reagent.

### Target prediction of miR-7641

The targets of miR-7641 were predicted using TargetScan (http://www.targetscan.org/vert_71/) algorithms. GO analyses of the predicted target genes for cellular component, molecular functions, biological processes, and protein classes were performed using the Panther (http://www.pantherdb.org/) database. Twelve potential targets genes were selected based on ‘context^++^ score’ and relevance of the genes to cancer.

### MiR-7641 target genes detection by qRT-PCR upon silencing with miR-7641-inhibitor

To validate the targets, miR-7641 expression was silenced using mirVana™ miRNA inhibitor (Catalog #4464084; Assay ID: MH29694; Thermo Fisher Scientific). Silencing efficacy was evaluated at 24, 48, and 72 h post-transfection. The ‘mir Vana™ miRNA Mimic negative Control’ was used as the experimental control. Isolation of mRNA and preparation of cDNA was carried out according to the manufacturer’s instructions provided with the Qiagen RNeasy kit (Qiagen, Hilden, Germany). The relative mRNA expressions of the 12 selected target genes were detected by qRT-PCR. Primer sets are shown in Supplementary Table S5.

### Luciferase activity assay

Luciferase activity assay was performed to confirm that *RPS16* and *TNFSF10* are real targets of miR-7641, the targeting sites of miR-7641 for *RPS16* (44–51 of *RPS16* 3′ UTR) and *TNFSF10* (924–931 & 932–939 of *TNFSF10* 3′ UTR) were separately inserted to the pmirGLO Dual-Luciferase miRNA Target Expression Vector (Cat. No. E1330; Promega Corporation, Madison, WI, USA). Then, the cloned vectors were transfected to the MCF-7 breast cancer cell lines along with either miR-7641 mimic (Catalog #: 4464066; Assay ID: MC29694; Thermo Fisher Scientific) or ‘mir Vana™ miRNA Mimic negative Control’. The luciferase activity was measured using Dual-Luciferase® Reporter Assay System at around 48 h post-transfection according to the manufacturer’s protocol. The ratio of firefly and renilla luciferase activity was calculated to determine the changes in luciferase activity in miR-7641 mimic transfected cells compared to ‘mir Vana™ miRNA Mimic negative Control’ transfected cells.

### qRT-PCR detection to analysis the mutually co-expressed genes of RPS16 and TNFSF10

For co-expression analysis, top 10 genes (*RPS5*, *RPS12*, *RPSA*, *RPL12*, *RPL18*, *RPL27A*, *RPL32*, *RPL38*, *EEF1A1* and *EEF1B2*) that are frequently co-expressed with *RPS16* and top five genes (*NCEH1*, *RARRES3*, *NTN4*, *CFB* and *CYP4Z1*) that are frequently co-expressed with *TNFSF10* in breast cancer patients, were selected for evaluation using qRT-PCR detection system. MCF-7 cells were transfected with either miR-7641 mimic or ‘mir Vana™ miRNA Mimic negative Control’ and the cells were harvested at around 48 h post-transfection. Isolation of mRNA and preparation of cDNA was carried out according to the manufacturer’s instructions provided with the Qiagen RNeasy kit (Qiagen, Hilden, Germany). The relative mRNA expressions of *RPS16* and *TNFSF10* along with their mutually co-expressed genes were detected by qRT-PCR. Primer sets are enlisted in Supplementary Table S5.

### In silico analysis using the TCGA database

The genetic alteration status and consequences of the alteration of *RPS16* and *TNFSF10*, along with their mutually co-expressed genes in breast cancer patients were analyzed using the cBioPortal for Cancer Genomics (http://www.cbioportal.org/index.do) database from TCGA Research Network (http://cancergenome.nih.gov/). For analysis, all samples (2509 patients/2509 samples) in “Breast Cancer (METABRIC, Nature 2012 & Nat Commun 2016)” were included. *RPS16* and its mutually co-expressed 10 genes were analyzed as a group (http://bit.ly/2torpCB), while *TNFSF10* with its mutually co-expressed five genes were considered as a separate group (http://bit.ly/2trjLr1). Patient’s survival status upon alteration of the selected genes, as well as networks and interplay of the selected genes with other genes that are potentially involved in breast cancer were also analyzed.

### Statistical analysis

Data obtained from triplicate experiments were analyzed statistically using one-way analysis of variance. The significance of the differences between control and treated cells was determined by Student’s *t* test. Significance was determined as *p* < 0.05, *p* < 0.01, and *p* < 0.001.

## Electronic supplementary material

Supplementary Information

Supplementary Spreadsheet
