# Supplementary material for: MicroRNA-7641 is a regulator of ribosomal proteins and a promising targeting factor to improve the efficacy of cancer therapy
Source: Sci Rep. 2017 Aug 21;7:8365. doi: 10.1038/s41598-017-08737-w (PMC5566380; doi:10.1038/s41598-017-08737-w)
Supplement: Supplementary file 1 — Supplementary Information [file 41598_2017_8737_MOESM1_ESM.pdf]

Supplementary Tables and Figures

MicroRNA-7641 is a regulator of ribosomal proteins and a promising targeting factor to improve the efficacy of cancer therapy

Abu Musa Md Talimur Reza, Yun-Jung Choi, Yu-Guo Yuan, Joydeep Das, Hideyo Yasuda & Jin-Hoi Kim*

Department of Stem Cell and Regenerative Biotechnology, Humanized Pig Research Centre (SRC), Konkuk University, Seoul 143-701, Republic of Korea

*** Corresponding author**: Department of Stem Cell and Regenerative Biotechnology, Humanized Pig Research Center (SRC), Konkuk University, Seoul 143-701, Republic of Korea.

E-mail address: jhkim541@konkuk.ac.kr ; *Tel:* +82-2-450-3687; *Fax: +82*-2-458-5414

Running title: MicroRNA-7641, ribosomal proteins and cancer

Supplementary Table S1. Predicted consequential pairing of target region (top) in different target genes and miRNA-7641 (bottom)

<table>
<tr><th rowspan="2">Target Gene</th><th colspan="4">Predicted target region (top) and miRNA (bottom)</th></tr>
<tr><th>Target Site-1</th><th>Target Site-2</th><th>Target Site-3</th><th>Target Site-4</th></tr>
<tr><td>RPS16</td><td>Position 44-51 of RPS16 3' UTR
5' ...GAGGCUUCCAAGAAGGAGAUCAA.
|||||||
3' CGAAUCGAAGGCUCUAGUU</td><td></td><td></td><td></td></tr>
<tr><td>RNF4</td><td>Position 34-40 of RNF4 3' UTR
5' ...CAACUUGUAGGAAAA-AGAUCAAC.
||| |||||
3' CGAAUCGAAGGCUCUAGUU</td><td>Position 151-158 of RNF4 3' UTR
5' ..UGCCUCCAUUUUCCUGAGAUCAA..
|||||||
3' CGAAUCGAAGGCUCUAGUU</td><td></td><td></td></tr>
<tr><td>TNFSF10</td><td>Position 924-931 of TNFSF10 3' UTR
5' ...AGGUGGGUAGAUCACGAGAUCAA..
||| ||||||
3' CGAAUCGAAGGCUCUAGUU</td><td>Position 932-939 of TNFSF10 3' UTR
5' ..AGAUCACGAGAUCAAGAGAUCAA..
|||||||
3' CGAAUCGAAGGCUCUAGUU</td><td>Position 1089-1095 of TNFSF10 3' UTR
5' ...AGGUUGCAGUGUGGUGAGAUCAU.
|||||||
3' CGAAUCGAAGGCUCUAGUU</td><td></td></tr>
<tr><td>ZNF616</td><td>Position 241-248 of ZNF616 3' UTR
5' ...AUCUUACUGGUAAAAGAGAUCAA...
|||||||
3' CGAAUCGAAGGCUCUAGUU</td><td>Position 966-972 of ZNF616 3' UTR
5' ..AAGGCAUUCAGACAUAGAUCAAA..
||||||
3' CGAAUCGAAGGCUCUAGUU</td><td>Position 3244-3250 of ZNF616 3' UTR
5' ...GCCAUAGAAAGGAAUGAGAUCAU.
|||||||
3' CGAAUCGAAGGCUCUAGUU</td><td></td></tr>
<tr><td>MSRB3</td><td>Position 180-186 of MSRB3 3' UTR
5' ...AUUUUGCAAUUGACUAGAUCAAG...
||||||
3' CGAAUCGAAGGCUCUAGUU</td><td>Position 901-907 of MSRB3 3' UTR
5' ..AGCUUGCAGUUAGCC---GAGAUCAU..
|||| ||||||
3' CGAAUCGAAGGCUCUAGUU</td><td>Position 1357-1363 of MSRB3 3' UTR
5' ...GAGAGAGAGCAGAGAAGAUCAAG.
||| |||||
3' CGAAUCGAAGGCUCUAGUU</td><td>Position 1676-1682 of MSRB3 3' UTR
5' ..UAUUGCUAUACAGUUGAGAUCAG..
|||||||
3' CGAAUCGAAGGCUCUAGUU</td></tr>
<tr><td>EMC8</td><td>Position 125-132 of EMC8 3' UTR
5' ...GACUGGACAAACCCAGAGAUCAA...
|||||||
3' CGAAUCGAAGGCUCUAGUU</td><td></td><td></td><td></td></tr>
<tr><td>CUL3</td><td>Position 128-135 of CUL3 3' UTR
5' ...AUGUUCUAGACCAUUGAGAUCAA...
||| |||||||
3' CGAAUCGAAGG-CUCUAGUU</td><td>Position 207-213 of CUL3 3' UTR
5' ..ACGUUUAUCCCUGUAGAGAUCAU..
|||||||
3' CGAAUCGAAGGCUCUAGUU</td><td></td><td></td></tr>
<tr><td>CDNF</td><td>Position 564-571 of CDNF 3' UTR
5' ..CUUAAAGAAAAUAGAGAGAUCAA...
|||||||
3' CGAAUCGAAGGCUCUAGUU</td><td>Position 1154-1160 of CDNF 3' UTR
5' ..UGAGUCUAGGAGUUAGAGAUCAG..
||| ||||||
3' CGAAUCGAAGG-CUCUAGUU</td><td></td><td></td></tr>
<tr><td>RAB29/
RAB7L1</td><td>Position 312-318 of RAB7L1 3' UTR
5' .AGCACCUGCGUUUUAGAGAUCAU..
|||||||
3' CGAAUCGAAGGCUCUAGUU</td><td>Position 395-402 of RAB7L1 3' UTR
5' .AAUUGAGAGGAAAAUGAGAUCAA.
|||||||
3' CGAAUCGAAGGCUCUAGUU</td><td>Position 845-851 of RAB7L1 3' UTR
5' .AGGUUGCAGUGAACCGAGAUCAU..
|||||||
3' CGAAUCGAAGGCUCUAGUU</td><td>Position 1589-1595 of RAB7L1 3' UTR
5' ...GAGGUCAGGAGUUCAAGAUCAAC
|||||||
3' CGAAUCGAAGGCUCUAGUU</td></tr>
<tr><td>SIGLEC7</td><td>Position 158-165 of SIGLEC7 3' UTR
5' ...AACAAUGAGUACUCAGAGAUCAA...
|||||||
3' CGAAUCGAAGGCUCUAGUU</td><td></td><td></td><td></td></tr>
<tr><td>PIGC</td><td>Position 116-123 of PIGC 3' UTR
5' ...UGUGGUAAGAGAAUAGAGAUCAA.
|||||||
3' CGAAUCGAAGGCUCUAGUU</td><td></td><td></td><td></td></tr>
<tr><td>NBEA</td><td>Position 1609-1615 of NBEA 3' UTR
5' ...AGUUCAUUUCAUCUUGAGAUCAU.
|||||||
3' CGAAUCGAAGGCUCUAGUU</td><td></td><td></td><td></td></tr>
</table>

Supplementary Table S2. Luciferase reporter assay data

RRS16						
	**NC**	**NC**	**NC**	**miR-7641**	**miR-7641**	**miR-7641**
Firefly luc activity	1860579	2039592	1730982	1528611	1624868	1528939
Renilla Luc activity	398439	433711	401701	506229	578241	699227
Firefly:Renilla	4.669670891	4.702652227	4.309130423	3.019603776	2.81001866	2.18661322
% Firefly:Renilla	99.99999766	100.0000048	100.0000098	64.66416534	59.7539146	50.7437283
Average			100.0000098			58.3872694
Standard Deviation			6.11009E-06			7.06012971
p value						0.00945922
**TNFSF10**						
	**NC**	**NC**	**NC**	**miR-7641**	**miR-7641**	**miR-7641**
Firefly luc activity	2948383	3417865	3368615	2109254	1922108	1518641
Renilla Luc activity	526880	581614	579292	431337	414778	330038
Firefly:Renilla	5.595928864	5.876517759	5.815055274	4.890037256	4.634064488	4.60141256
% Firefly:Renilla	100	100	100	87.38562221	78.85732125	79.1293005
Average			100			81.790748
Standard Deviation			1.47431E-09			4.84721118
p value						0.02281471

Supplementary Table S3. The table contains genes that are frequently co-expressed with *RPS16* in breast cancer patients, with the corresponding details.

Correlated Gene	Cytoband	Pearson's Correlation	Spearman's Correlation
RPL27A	11p15	0.76	0.71
RPS5	19q13.4	0.74	0.68
RPS12	6q23.2	0.73	0.65
RPL38	17q25.1	0.71	0.65
RPL12	9q34	0.69	0.63
EEF1A1	6q14.1	0.68	0.54
RPL32	3p25-p24	0.67	0.58
RPSA	3p22.2	0.67	0.6
EEF1B2	2q33.3	0.67	0.56
RPL18	19q13	0.67	0.59
RPL30	8q22	0.66	0.56
RPL27	17q21	0.66	0.61
RPS17	15q	0.66	0.57
RPL13AP3	14q22.3	0.66	0.68
RPLP0	12q24.2	0.65	0.54
RPL35A	3q29	0.63	0.55
RPS3	11q13.3-q13.5	0.63	0.58
RPL18A	19p13	0.62	0.54
SLC25A5	Xq24	0.6	0.43
RPS15A	16p	0.6	0.51
HNRNPA1L2	13q14.3	0.6	0.47
RPL24	3q12	0.59	0.52
EEF1G	11q12.3	0.59	0.48
RPS9	19q13.4	0.58	0.53
RPL17	18q21	0.58	0.5
RPS27A	2p16	0.57	0.44
COMMD6	13q22	0.57	0.44
HINT1	5q31.2	0.56	0.4
ATP5O	21q22.11	0.56	0.4
UQCRFS1	19q12	0.56	0.39
TAOK2	16p11.2	-0.53	-0.41
SLC38A7	16q21	-0.48	-0.32
FAM214B	9p13.3	-0.45	-0.37
MICALL2	7p22.3	-0.45	-0.35
ZNF76	6p21.31	-0.45	-0.3
TMEM175	4p16.3	-0.45	-0.37
FICD	12q24.1	-0.45	-0.4
GRIPAP1	Xp11.23	-0.44	-0.3
TTC14	3q26.33	-0.43	-0.32
PIP5K1C	19p13.3	-0.43	-0.36
RFX1	19p13.1	-0.43	-0.32
SIL1	5q31	-0.42	-0.37
VPS18	15q15.1	-0.42	-0.33
DCTN1	2p13	-0.41	-0.34
TOM1	22q13.1	-0.41	-0.34
GNA11	19p13.3	-0.41	-0.31
MBD6		-0.41	-0.34
IGSF8	1q23.1	-0.4	-0.33
PLPPR2	19p13.2	-0.4	-0.35
ADCK1	14q24.3	-0.4	-0.31
ADCY6	12q13.12	-0.4	-0.32
NFIC	19p13.3	-0.39	-0.31
CAMSAP3	19p13.2	-0.39	-0.32
ARAF	Xp11.4-p11.2	-0.38	-0.3
RRBP1	20p12	-0.38	-0.31
NAGLU	17q21	-0.38	-0.37
PLBD2	12q24.13	-0.38	-0.34
RGL2	6p21.3	-0.37	-0.31
CHPF	2q35	-0.37	-0.34
ENTPD5	14q24	-0.37	-0.33

Supplementary Table S4. The table contains genes that are frequently co-expressed with *TNFSF10* in breast cancer patients, with the corresponding details.

Correlated Gene	Cytoband	Pearson's Correlation	Spearman's Correlation
NCEH1	3q26.31	0.46	0.46
RARRES3	11q23	0.42	0.37
CFB	6p21.3	0.41	0.38
NTN4	12q22	0.41	0.39
LTF	3p21.31	0.39	0.42
CYP4Z1	1p33	0.39	0.4
PIP	7q34	0.37	0.36
CYP4X1	1p33|1	0.37	0.37
LRRC26	9q34.3	0.36	0.35
NOSTRIN	2q31.1	0.36	0.35
HMGCS2	1p13-p12	0.35	0.34
SLC44A4	6p21.3	0.34	0.34
LRRC31	3q26.2	0.34	0.37
CYBRD1	2q31.1	0.34	0.33
MUC1	1q21	0.34	0.33
CYP4Z2P	1p33	0.34	0.36
IQGAP2	5q13.3	0.33	0.32
TC2N	14q32.12	0.33	0.32
IL13RA1	Xq24	0.32	0.31
SHROOM1	5q31.1	0.32	0.33
ARHGDIB	12p12.3	0.32	0.32
CCDC160	Xq26.2	0.31	0.33
GPC4	Xq26.1	0.31	0.31
TACSTD2	1p32	0.31	0.31
TRIM5	11p15	0.31	0.31
CD46	1q32	0.3	0.31
UBE2S	19q13.43	-0.37	-0.37
PRELID3A	18p11.21	-0.37	-0.37
FAM216A	12q24.11	-0.37	-0.35
TOMM40	19q13	-0.35	-0.35
FAM64A	17p13.2	-0.35	-0.33
NETO2	16q11	-0.35	-0.34
PBK	8p21.2	-0.34	-0.33
SCRIB	8q24.3	-0.33	-0.33
PTDSS1	8q22	-0.33	-0.33
CCNB1	5q12	-0.33	-0.32
RECQL4	8q24.3	-0.32	-0.31
NCAPG	4p15.33	-0.32	-0.31
AURKA	20q13	-0.32	-0.31
PSRC1	1p13.3	-0.32	-0.31
PGAM5	12q24.33	-0.32	-0.33
E2F7	12q21.2	-0.32	-0.31
TROAP	12q13.12	-0.32	-0.31
TIGD5	8q24.3	-0.31	-0.31
PUF60	8q24.3	-0.31	-0.3
MTERF3	8q22.1	-0.31	-0.31
RAD54B	8q22.1	-0.31	-0.31
TMEM70	8q21.11	-0.31	-0.31
TRIP13	5p15.33	-0.31	-0.32
UBE2C	20q13.12	-0.31	-0.3
MCM10	10p13	-0.31	-0.31
TGIF2	20q11.23	-0.3	-0.3
CDC20	1p34.1	-0.3	-0.3
KIF2C	1p34.1	-0.3	-0.3

Supplementary Table S5. List of primers used for the experiments

**A. Primers used to confirm miRNA expression**		
**miRNA Name**	**miRNA specific 5' primer**	**mRQ 3' Primer**
hsa-miR-4792	5′-CGGTAGCGCTCGCTGGC-3′	Provided with Mir-X™ miRNA qRT-PCR SYBR® Kit (Clontech Laboratories, Inc., Takara Bio, CA, US)
hsa-miR-7704	5′-CGGGGTCGGCGGCGACGTG-3′	
hsa-miR-6087	5′-TGAGGCGGGGGGGCGAGC-3′	
hsa-miR-4466	5′-GGGTGCGGGCCGGCGGGG-3′	
hsa-miR-4532	5′-CCCCGGGGAGCCCGGCG-3′	
hsa-miR-7641	5′-TTGATCTCGGAAGCTAAGC-3′	
hsa-miR-4448	5′-GGCTCCTTGGTCTAGGGGTA-3′	
hsa-miR-3960	5′-GGCGGCGGCGGAGGCGGGGG-3′	
hsa-miR-1246	5′-AATGGATTTTTGGAGCAGG-3′	
hsa-miR-3687	5′-CCCGGACAGGCGTTCGTGCGACGT-3′	
**B. Primers used to detect target molecule transcript (mRNA) expression**		
**Gene**	**Forward Primer**	**Reverse Primer**
RAB29/RAB7L1	5′-AAGCTCACACTACCCAATGG-3′	5′-ACTGATGTTTCTGTCCAACCTG-3′
TNFSF10	5′-CAGCTCACATAACTGGGACC-3′	5′-CCATTCCTCAAGTGCAAGTTG-3′
CUL3	5′-AGGTTCTACTTAGCCAAACACAG-3′	5′-ACCTCCAACACCAACTTCAG-3′
SIGLEC7	5′-GTGAACGTGACAGAGTGAGG-3′	5′-GTTATCATCTGCCCAGGACTC-3′
ZNF616	5′-AAAATACCCATAGCCCCTTCG-3′	5′-AGCCATCACTGACTCCTTTTC-3′
RNF4	5′-GCACCAAAGAGCACAATGAG-3′	5′-GAGTTCTATGGGTTCTGCTTCC-3′
RPS16	5′-GTGTCCGTGTAAAGGGTGG-3′	5′-ACTGGATGAGGATGTCTTTGATC-3′
PIGC	5′-GCAACCCTAGGAACTCTCG-3′	5′-CCATGCTGTGTTGATGTTCTAC-3′
MSRB3	5′-GAGGCAATCACATTCACAGATG-3′	5′-GACAAGGCAGCCGAATTTATG-3′
CDNF	5′-TGAAGTCACTCGCCCAATG-3′	5′-GCTCTGCCACTCTCATCTTC-3′
EMC8	5′-ATGCCAGTCCAAACCAGG-3′	5′-ATCTGTTCTCATGGTGCTCG-3′
NBEA	5′-TGGTGATATGGCTTGGCATG-3′	5′-ATATCTGTGCTGGGTTGAGTG-3′
**C. Primers used to detect the genes co-expressed with RPS16 and TNFSF10**		
**Gene**	**Forward Primer**	**Reverse Primer**
RPL27A	5′-CCACCGGATCAACTTCGAC-3′	5′-TTCACCCGTGTCTGTTCAC-3′
RPS5	5′-CAGAGACCCCAGACATCAAG-3′	5′-TTGGCATACTTCTCCTTCACTG-3′
RPS12	5′-GAAACTAGGAGAATGGGTAGGC-3′	5′-AATGACATCCTTGGCCTGAG-3′
RPL38	5′-GCCATGCCTCGGAAAATTG-3′	5′-CCAGGGTGTAAAGGTATCTGC-3′
RPL12	5′-GGAACCACCAAGAGACAGAAAG-3′	5′-GTCCCCAGGATCTCTTTAATGG-3′
EEF1A1	5′-TCATTGGACACGTAGATTCGG-3′	5′-AAGACCCAGGCATACTTGAAG-3′
RPL32	5′-AACGTCAAGGAGCTGGAAG-3′	5′-GGGTTGGTGACTCTGATGG-3′
RPSA	5′-TTGCCATTGAAAACCCTGC-3′	5′-GCCTGGATCTGGTTAGTGAAG-3′
EEF1B2	5′-AGTATTTGAAGCCGTGTCCAG-3′	5′-ACATCGGCAGGACCATATTTG-3′
RPL18	5′-TGGACATCCGCCATAACAAG-3′	5′-CAACCTCTTCAACACAACCTG-3′
NCEH1	5′-CCCGAAGAGCCACTGAAAC-3′	5′-GACAGCATTCAATTCCTCAGC-3′
RARRES3	5′-ATCAGTTCTGCGAAGGAGATG-3′	5′-ACACCGACTTCAACCTTGG-3′
CFB	5′-ACCAAAAGACTGTCAGGAAGG-3′	5′-GAGAGTGTAACCGTCATAGCAG-3′
NTN4	5′-ACTGCTCCGCTACATTTGG-3′	5′-GGGTTCTCTGTATCGTATGGTG-3′
CYP4Z1	5′-ACCCTGGATGGTTCTAAATGG-3′	5′-CGTGAGTTTTGGGCAATGTG-3′

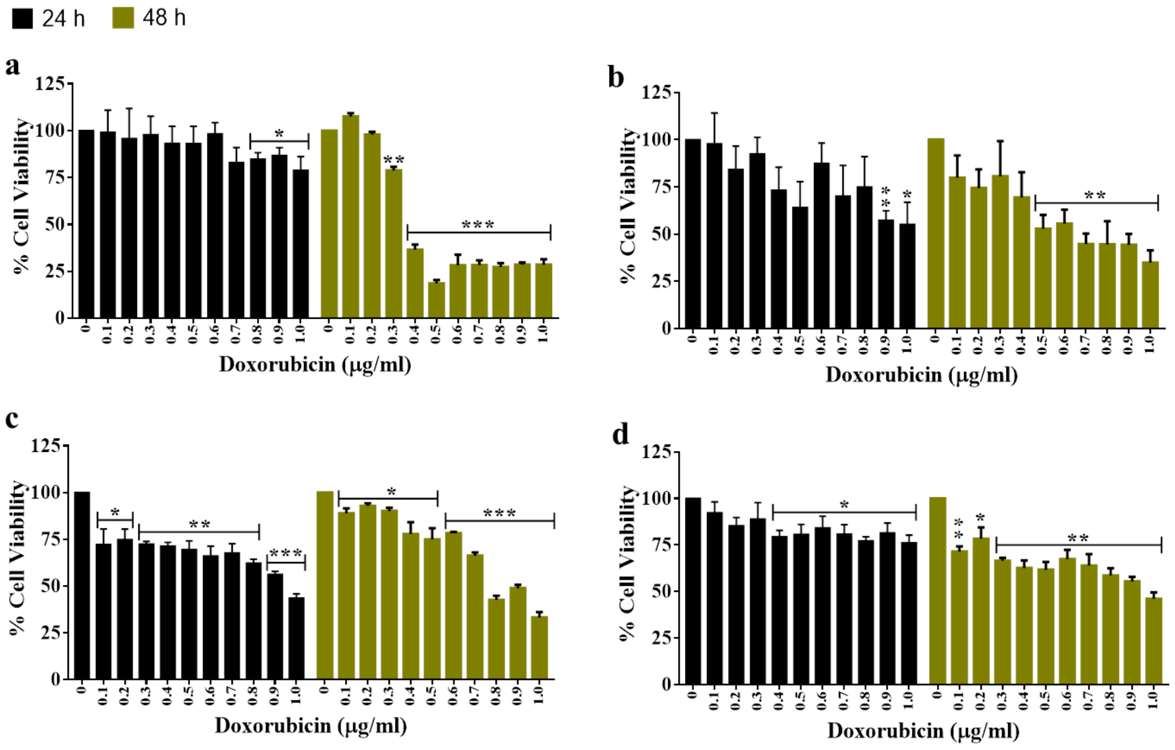

Supplementary Figure S1. Dose- and time-dependent effects of doxorubicin in different cancer cell lines. **a,** Dose- and time-dependent influence of doxorubicin on MCF-7 breast cancer cells. **b,** Dose- and time-dependent effects of doxorubicin on MDA-MB-231 breast cancer cells. **c,** Dose- and time-dependent effects of doxorubicin on HT-29 colon cancer cells. **d,** Dose- and time-dependent influence of doxorubicin on HCT116 colon cancer cells. Bar diagrams presenting the mean ± SD obtained from triplicate experiments. *$p < 0.05$, **$p < 0.01$, and ***$p < 0.001$.

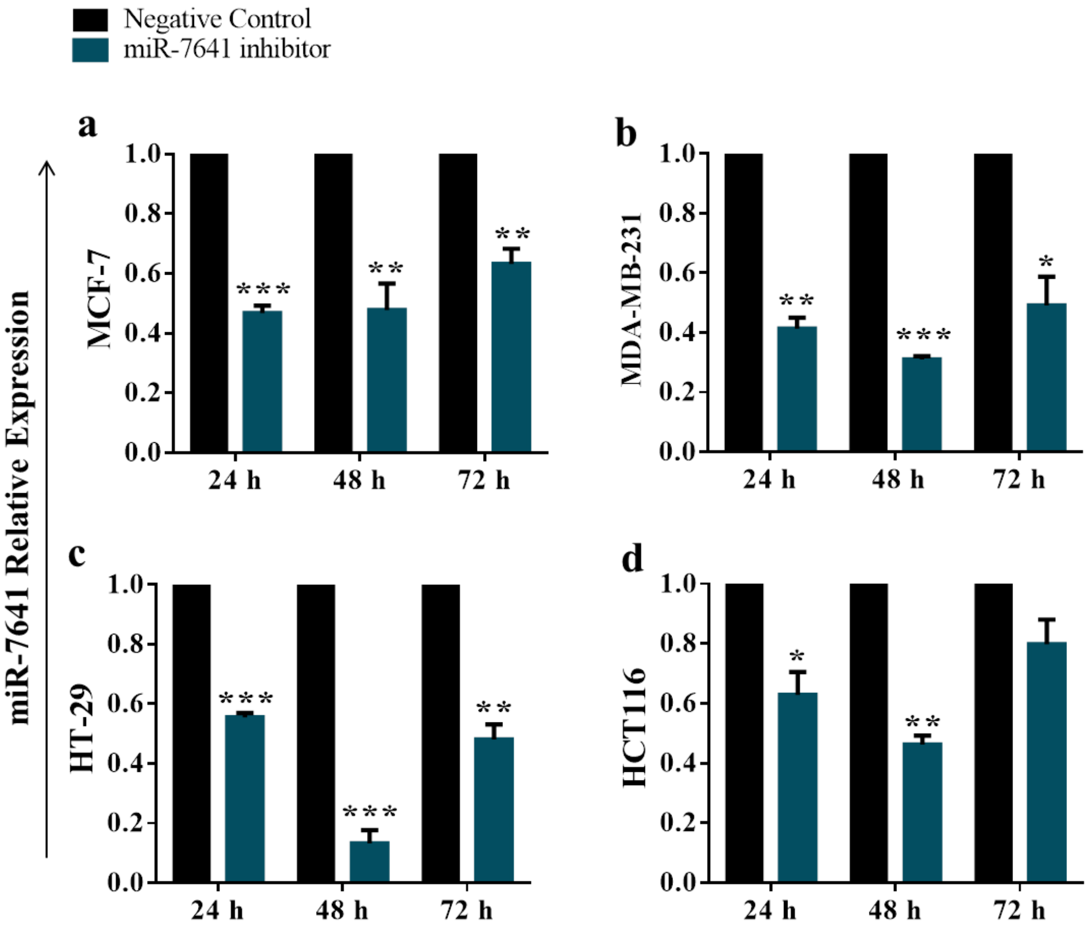

Supplementary Figure S2. Efficiency of miR-7641 inhibitor at different time points in different cell lines. **a,** miR-7641 inhibitor showed highest efficiency between 24 h and 48 h post-transfection in MCF-7 breast cancer cells. **b,** miR-7641 was most effective at 48 h post-transfection in MDA-MB-231 breast cancer cells. **c,** miR-7641 was most effective at 48 h post-transfection in HT-29 colon cancer cells. **d,** miR-7641 was most effective in 48 h post-transfection in HCT116 colon cancer cells. Bar diagrams presenting the mean ± SD obtained from triplicate experiments. *$p < 0.05$, **$p < 0.01$, and ***$p < 0.001$.

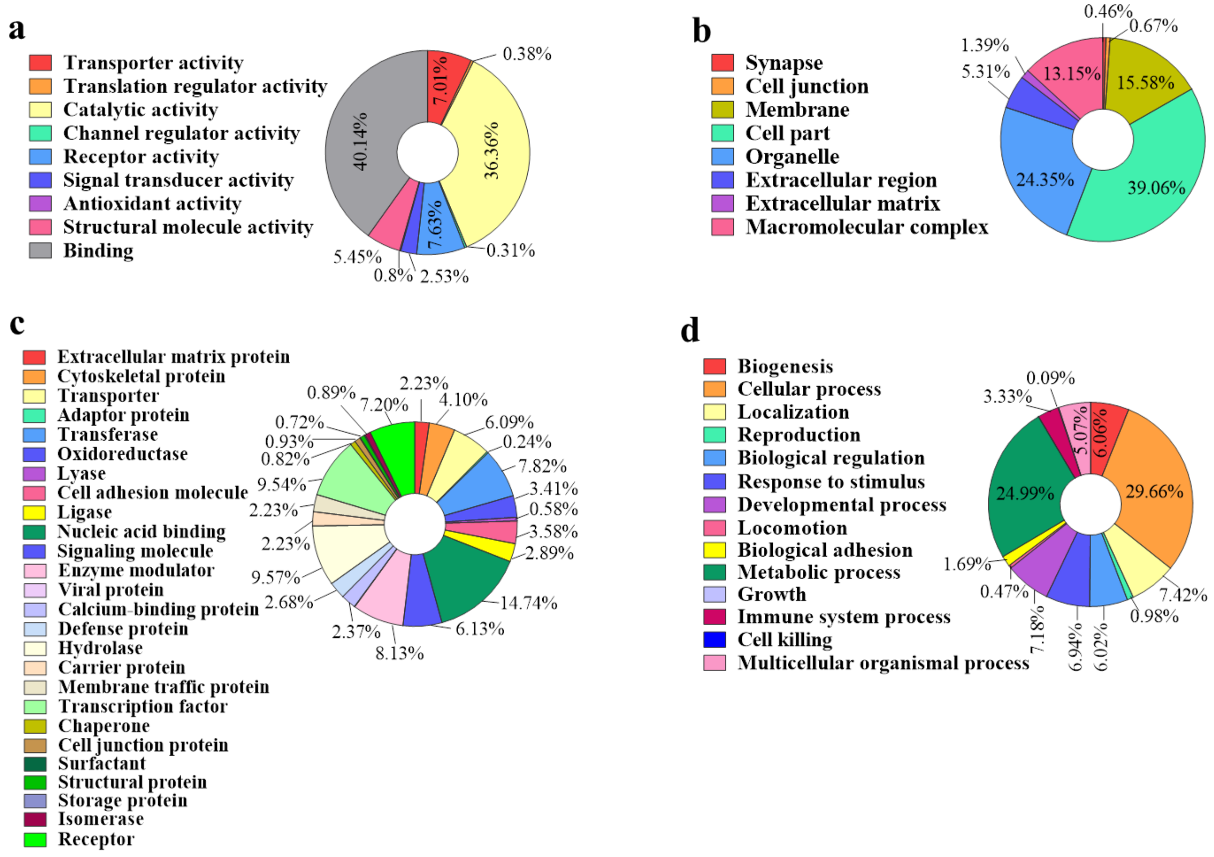

Supplementary Figure S3. *In silico* GO analyses of miR-7641 target genes using the PANTHER classification system. **a,** GO molecular function analysis indicated that more than 40 % were binding molecules and around 36 % were involved in catalytic activity. **b,** GO cellular component analysis showed an association of 39 % of the molecules with cell part, 24 % with organelles, and 15 % with membranes. **c,** GO terms associated with protein classes were diverse, with around 15 % of the target molecules being nucleic acid binding factors, 10 % transcription factors, 10 % hydrolases, 8 % enzyme modulators, and 7 % receptors. **d,** GO biological processes analysis showed that around 30 % of the molecules were involved in cellular processes and 25 % were involved in metabolic processes. The target genes were predicted using TargetScan software, and the GO analysis was performed using the PANTHER classification system.

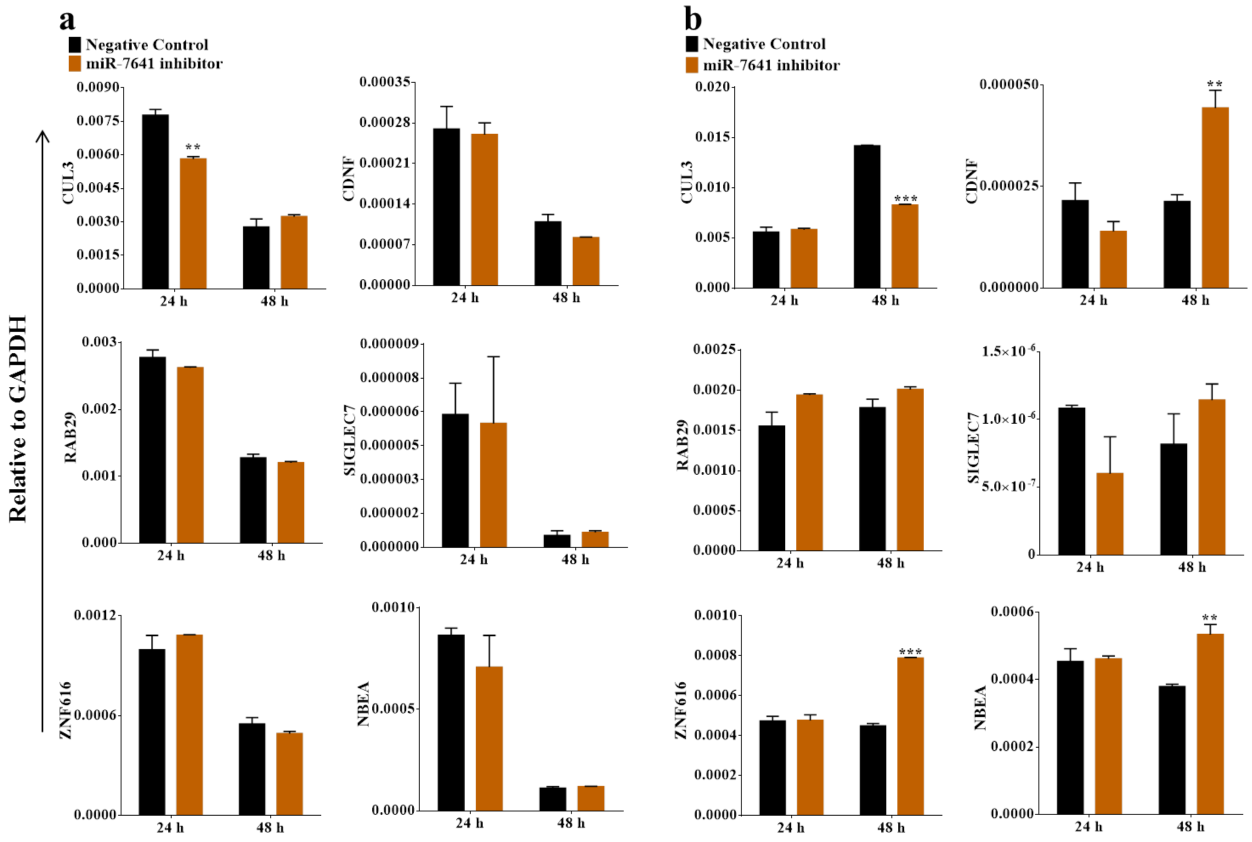

Supplementary Figure S4. Inhibition of miR-7641 altered target gene expression in breast cancer cells. **a,** Bar diagrams showing that inhibition of miR-7641 downregulated the expressions of *CUL3* in MCF-7 breast cancer cells. **b,** As shown in the bar diagrams, inhibition of miR-7641 downregulated the expression of *CUL3* and upregulated the expressions of *CDNF*, *ZNF616* and *NBEA* in MDA-MB-231 breast cancer cells. Bar diagrams show the average ± SD obtained from triplicate experiments. *$p < 0.05$, **$p < 0.01$, and ***$p < 0.001$.

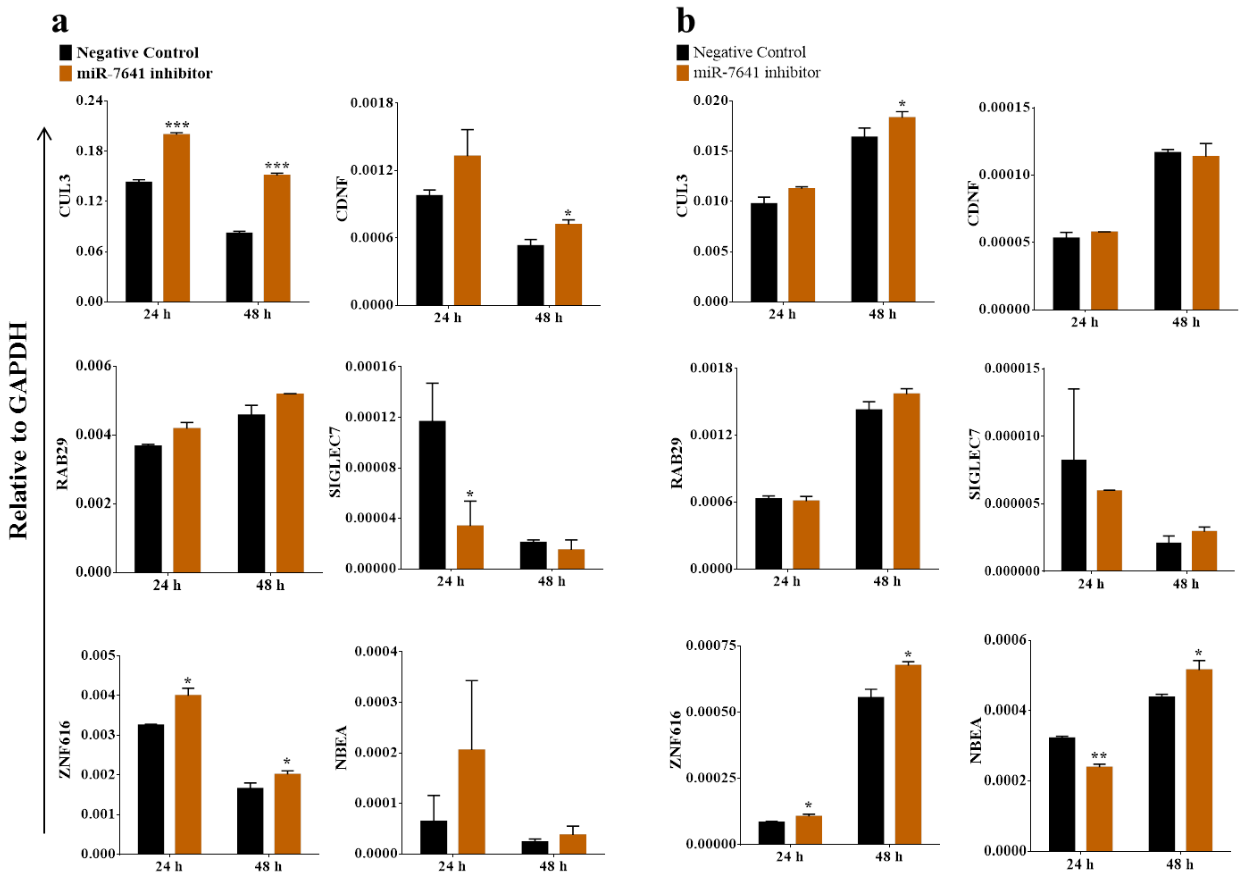

Supplementary Figure S5. Inhibition of miR-7641 altered target gene expression in colon cancer cells. **a,** Bar diagrams presenting the expression patterns of miR-7641 target genes in HT-29 colon cancer cells upon transfection with a locked nucleic acid inhibitor against miR-7641. Inhibition of miR-7641 resulted in the upregulation of *CUL3* and *CDNF* expression. **b,** Bar diagrams showing that inhibition of miR-7641 upregulated *CUL3*, *ZNF616* and *NBEA* expression in HCT116 colon cancer cells. Bar diagrams show the mean ± SD obtained from triplicate experiments. *$p < 0.05$, **$p < 0.01$, and ***$p < 0.001$.

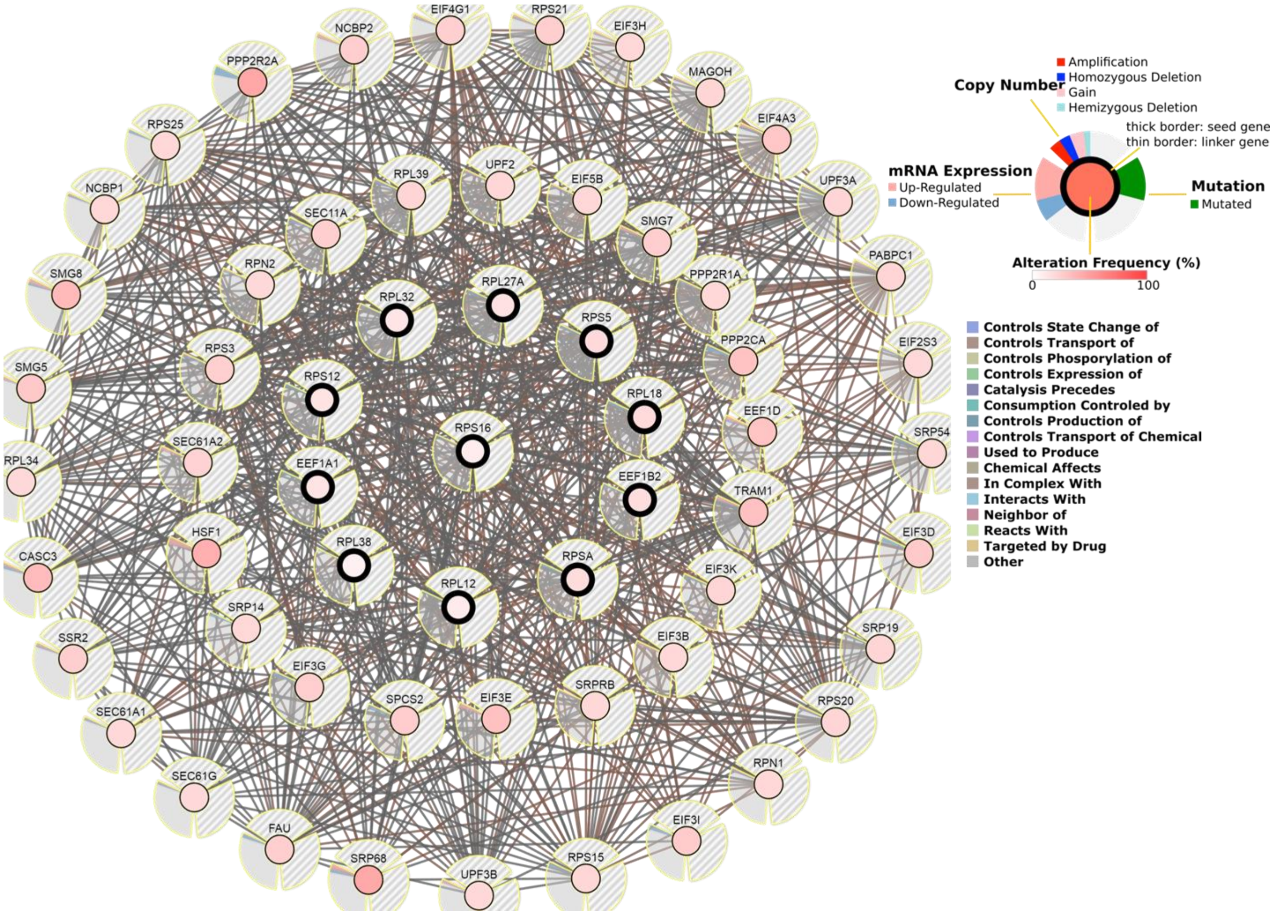

Supplementary Figure S6. Networks of miR-7641 target gene *RPS16* with other genes involved in breast cancers. The data were generated by the TCGA Research Network: http://cancergenome.nih.gov/."
